# Enhancing Magnesium-Ion Storage in a Bi–Sn Anode through Dual-Phase Engineering

**DOI:** 10.1021/acsami.4c11272

**Published:** 2024-10-05

**Authors:** Muhammad Rashad, Apinya Ngoipala, Matthias Vandichel, Hugh Geaney

**Affiliations:** Department of Chemical Sciences and Bernal Institute, University of Limerick, Limerick V94 T9PX, Ireland

**Keywords:** Mg-ion batteries, alloying mode anodes, bismuth/tin active materials, dual-phase engineering, DFT calculations, electrochemical characterization

## Abstract

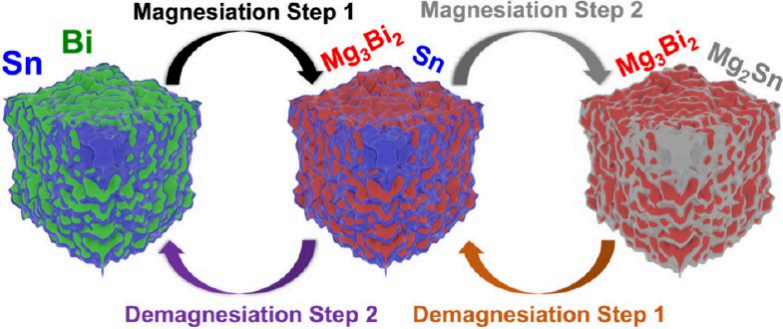

Magnesium-ion batteries (MIBs) are a “beyond Li-ion” technology that are hampered by Mg metal reactivity, which motivates the development of anode materials such as tin (Sn) with high theoretical capacity (903 mAh g^–1^). However, pure Sn is inactive for Mg^2+^ storage. Herein, Mg alloying with Sn is enabled within dual-phase Bi–Sn anodes, where the optimal composition (Bi_66.5_Sn_33.5_) outperformed single-phase Bi and Sn electrodes to deliver high specific capacity (462 mAh g^–1^ at 100 mA g^–1^), good cycle life (84% after 200 cycles), and significantly improved rate capability (403 mAh g^–1^ at 1000 mA g^–1^). Density functional theory (DFT) calculations revealed that Mg alloys first with Bi and the subsequent formation of the Mg_3_Bi_2_//Sn interfaces is energetically more favorable compared to the individual Mg_3_Bi_2_ and Sn phases. Mg insertion into Sn is facilitated when Mg_3_Bi_2_ is present. Moreover, dealloying Mg from Mg_3_Bi_2_:Mg_2_Sn systems requires the creation of Mg vacancies and subsequent Mg diffusion. Mg vacancy creation is easier for Mg_2_Sn compared to Mg_3_Bi_2_, while the latter has slightly lower activated Mg-diffusion pathways. The computational findings point toward easier magnesiation/demagnesiation for BiSn alloys over pure Bi or pure Sn, corroborating the superior Mg storage performance of Bi–Sn electrodes over the corresponding single-phase electrodes.

## Introduction

Mg-ion batteries (MIBs) have emerged as one of the most attractive post-Li-ion technologies due to potential advantages over lithium-ion batteries (LIBs),^1−5^ including high volumetric capacity (3833 mAh·cm^–3^), high natural abundance of Mg (8th), and the divalent nature of Mg^2+^.^6−8^ Despite these advantages, the development of MIBs is restricted by several hurdles, including the availability of compatible cathode materials and electrolytes.^9^ Mg metal is incompatible with conventional electrolytes including Mg(ClO_4_)_2_, Mg(BF_4_)_2_, Mg(PF_6_)_2_, and Mg(TFSI)_2_ in carbonate or nitrile-based solvents, due to the formation of a passivation layer, which subsequently blocks Mg^2+^ ion transport.^10^ Only ether-based electrolytes (i.e., Grignard reagents or borohydride-based electrolytes) have shown promising compatibility with the Mg metal anodes. However, their low anodic stability, complex preparation procedures, high sensitivity to water, corrosive nature, and low Coulombic efficiencies remain challenging.^11,12^ Thus, substituting Mg metal anodes with Mg alloy forming active materials may bypass the passivation issue and enable the use of more conventional electrolytes. Alloying-type anode materials have high theoretical capacities and low working potentials when compared with insertion-type materials.^13,14^ Thus, the combination of low voltage alloying-type anodes with high voltage compatible cathodes and conventional electrolytes is the most promising route for the development of MIBs with high energy densities.^15^

Recent research on alloying-type anodes for MIBs has focused on bismuth (Bi),^16,17^ tin (Sn),^18,19^ antimony (Sb),^20^ indium (In),^21^ and gallium (Ga).^22^ However, the reversible capacities of these materials are either limited due to sluggish Mg^2+^ ion transportation in the host lattices or rapid decay due to material volume expansion during solid–solid phase transformations.^23^ To circumvent these issues, several tactics have been employed, including preparing graphene composites,^16^ decreasing particle size,^24^ designing amorphous or 3D structures,^25^ doping with foreign elements, and dual-phase engineering.^26^ Among these strategies, dual-phase engineering (by combining different p-block elements) is a powerful method as it unlocks synergetic effects of the different materials.^27^ To date, several dual-phase anode materials have been investigated including Bi–In,^28^ Bi–Pb,^29^ Sn–Ga,^26^ In–Sb,^30^ In–Pb,^31^ Sn–Sb,^27^ and Bi–Sn.^32^ These dual-phase materials show significant improvements in electrochemical properties; however, they are still restricted due to their limited capacities, complex synthetic procedures, and limited understanding of the magnesiation/demagnesiation processes occurring. Combining highly active Bi with group IV-A elements (i.e., Pb, Sn, Ge, or Si) can be beneficial in terms of achievable specific capacities and stability of the anodes. Theoretical and experimental works have proven that the diffusivity of divalent Mg^2+^ ion inside Bi is similar to that of monovalent Li^+^ ion (10^–14^ cm^2^ s^–1^), making it an ideal anode material.^33^ However, the specific capacity of Bi is limited to 375 mAh g^–1^ and it also has the issue of fast capacity fading.^17^ In contrast, group 14 elements have high theoretical capacities, i.e., 517, 903, 1476, and 3817 mAh g^–1^, when forming Mg_2_Pb,^34^ Mg_2_Sn,^35^ Mg_2_Ge,^15^ and Mg_2_Si,^36^ respectively. However, these theoretical capacities are not achievable due to the high insertion barrier of Mg^2+^ into the host lattices, rendering them inactive for Mg^2+^ storage.^15,36^ Therefore, combining highly active Bi with less active Pb, Sn, Ge, or Si matrices may reduce the defect formation energy of Mg^2+^ ion insertion, allowing these materials to become electrochemically active.^32^ Recently, Song et al.^29^ synthesized Bi–Pb alloying anodes using a sputtering method, which allowed significant improvement in delivered capacities compared with pure Bi and Pb. However, the maximum capacity of the Bi–Pb anode was limited to 339 mAh g^–1^ at 20 mA g^–1^ with about 60% capacity retention. Compared to Pb, Sn has a higher theoretical capacity (903 mAh g^–1^) and lower diffusion barrier than Ge or Si;^36^ thus it is an ideal candidate for Bi–Sn dual-phase anodes. In another work, Song et al.^32^ synthesized the Bi_33_Sn_67_ dual-phase anode using the RF magnetron sputtering method; however the maximum reversible capacity was limited to 350 mAh g^–1^ with poor cycle life, suggesting that Bi_*x*_Sn_*y*_ compounds are promising systems for Mg-ion applications but require additional optimization to deliver improved electrochemical properties and stabilities.

Herein, we employ a dual-phase engineering strategy for Bi–Sn anodes for MIBs by using thermal evaporation. Thermal evaporation allows for precise control over the deposition process, leading to a uniform distribution of the Bi and Sn phases. This uniformity is harder to achieve by other approaches’ simple physical mixture method, which can result in phase segregation and nonuniform performance. The introduction of crystalline Bi alongside Sn reduces the defect formation energy of Mg^2+^ ion insertion, which ultimately improves the electrochemical activity of the Sn. Our results reveal that compared to single-phase Bi_100_ or Sn_100,_ the Bi_66.5_Sn_33.5_ dual-phase electrode delivers an excellent cycle life and rate capability for Mg^2+^ storage due to synergistic effects of the interspersed Bi and Sn domains. The Bi enables activation of the Sn, thus increasing the reversible capacity, while the low lattice expansion of Sn helps to stabilize the electrode active material during phase transformations. The dual-phase approach ensures a consistent interface between Bi and Sn, which is crucial for improving electrochemical performance.

With the help of experimental results and extensive computational density functional theory (DFT) calculations, we explain this mechanism, whereby the presence of Bi boosts the reversibility of Mg^2+^ alloying in the Sn. The Mg^2+^ storage mechanism in single-phase Bi and Bi–Sn dual-phase is revealed using ex situ XRD and TEM at different states of charge and discharge.

## Experimental Procedures

### Electrode Preparation

Dendritic copper foils (with purity of 99.9%) were cleaned using ultrasonication in acetone for 1 h followed by drying in a vacuum oven. Cleaned foils were transferred into a Mbraun, MB-200B glovebox fitted with a thermal evaporator. Foils were loaded into the evaporator chamber containing Bi or Sn billets (with purity of 99.9%, supplied by Kurt. J. Lesker Company, Germany). A vacuum of 3 × 10^–5^ Torr was achieved, followed by Bi or Sn deposition for 20 min to achieve an average loading of 0.2 mg·cm^–2^ onto dendritic copper foils. For Bi–Sn dual-phase samples, sequential evaporation of Sn layers sandwiched between Bi layers was used to obtain Bi_66.5_Sn_33.5_ and Bi_50_Sn_50_ by weight percentage. During thermal evaporation, we carefully controlled the deposition parameters, including the deposition rate, substrate temperature, and deposition time, to achieve a uniform thickness and composition. The deposition rate for each material was optimized to ensure that Bi and Sn were deposited in the desired ratios, maintaining the dual-phase structure throughout the electrode. The Bi_100_, Sn_100,_ Bi_66.5_Sn_33.5_, and Bi_50_Sn_50_ electrodes were stored in the Ar-filled glovebox before cell assembly.

### Materials Characterization

An ultramicrobalance (SE2) (repeatability ±0.25 μg) was used to measure the weight of active materials (Bi, Sn, or Bi–Sn) deposited onto the Cu current collectors. The electrodes were characterized using the X-ray diffraction (XRD) technique with a PANalytical Empyrean diffractometer having a PIXcel detector, Cu Kα radiation source, wavelength of 1.5301 Å, operating potential, and current of 40 kV and 40 mA, respectively. The morphologies of the deposited Bi or Sn layers were investigated using a scanning electron microscope (SEM; Hitachi SU-70) equipped with an EDS detector operating between 5 and 15 kV. The EDS detector was used to measure the elemental composition and distribution of the deposited Bi–Sn layers. For transmission electron microscopy (TEM) analysis, samples were ultrasonicated in 2-propanol and drop casted onto Cu grids with a lacey carbon layer. Casted grids were dried in vacuum of about 1 × 10^–7^ Torr for 12 h to remove the organic residues. Finally, TEM (JEOL JEM-2100F) equipped with a Gatan Ultrascan CCD camera was used to examine the Bi–Sn dual-phase anodes.

### Electrochemical Characterization

Half-cells (CR2032) were assembled in an argon-filled glovebox using Mg foil as a counter and reference electrode, a Bi, Sn, or Bi–Sn electrode as working electrode, a glass microfiber separator (Whatman GF/A, China), and 0.5 M Phenyl Mg chloride in tetrahydrofuran (supplied by Sigma-Aldrich, Ireland) as the electrolyte. All cells were aged for about 3 h before starting a galvanostatic test. Galvanostatic charging–discharging tests were conducted using a potential window of 0.05–0.6 V at the current densities of 100, 200, 400, 600, 800, 1000 mA g^–1^ using a Neware BTS 8.0 station, China. Apart from charging–discharging tests, cyclic voltammetry (CV) was also carried out between 0.05 and 1.0 V using a Biologic MPG-2 instrument. Electrochemical impedance spectroscopy (EIS) of half-cells was carried out before and after different numbers of cycles (at the current density of 200 mA g^–1^) using a frequency range of 0.1 Hz to 10 kHz at room temperature. The post-mortem of electrodes in different charged and discharged states was carried out using ex situ XRD and TEM. The scanning TEM images and elemental mapping were obtained using TEM (JEOL JEM-2100F) in a high-angle annular dark field (HAADF) mode.

### Computational Details

Periodic density functional theory (DFT) calculations was carried out in the Vienna Ab Initio Simulation Package (VASP 6.1.2)^37−39^ employing the generalized gradient approximation (GGA) functional of Perdew, Burke, and Ernzerhof (PBE)^40^ to describe the exchange–correlation. The projector-augmented-wave (PAW) method^41,42^ was used to describe the electron–ionic core interaction, where Sn 4d^10^5s^2^5p^2^, Bi 5d^10^6d^2^6p^3^, and Mg 2p^6^3s^2^ were treated as valence electrons. The cutoff energy for the plane-wave basis was set to 500 eV for the structural relaxations. The unit cells of all structures were optimized using the conjugate gradient method^43^ (Table 1). The Methfessel–Paxton smearing technique^44^ (σ = 0.2 eV) was used during the geometry optimization. All structures were fully relaxed until the force acting on each atom was less than 0.01 eV/Å and the convergence tolerance was set to 1 × 10^–6^ eV.

**Table 1:** Calculated Unit Cell Parameters for the Considered Materials with Their Space Group Are Compared with the Experimental Values

		calculated values (this work)				experimental values (this work)			
materials	space group	*a* (Å)	*b* (Å)	*c* (Å)	α, β, γ (deg)	*a* (Å)	*b* (Å)	*c* (Å)	α, β, γ (deg)
Sn	*I*4_1_/*amd*	5.827	5.827	3.324	90, 90, 90	5.819	5.819	3.175	90, 90, 90
Bi	*R*3̅*m*	4.576	4.576	12.129	90, 90, 120	4.557	4.557	11.862	90, 90, 120
Mg_2_Sn	*Fm*3̅*m*	6.820	6.820	6.820	90, 90, 90	6.759	6.759	6.759	90, 90, 90
Mg_3_Bi_2_	*P*3̅*m*1	4.708	4.708	7.456	90, 90, 120	4.671	4.671	7.403	90, 90, 120

The unit cells of β-Sn, Mg_2_Sn, Bi, and Mg_3_Bi_2_ were first optimized, as depicted in Figure S11. To study the mechanism of Mg diffusion, a 2 × 2 × 2 supercell of Mg_2_Sn and 3 × 3 × 2 supercell of Mg_3_Bi_2_ were constructed as shown in Figure S12. To obtain the diffusion barriers, we adopted the climbing image nudged elastic band (CI-NEB) method^45^ to determine the minimum energy path of ion migration, with eight images along the CI-NEB path connecting between two local minima. Different active planes (*hkl*) for Sn, Bi, Mg_2_Sn, and Mg_3_Bi_2_ were detected from XRD characterizations in the present work, as listed in Table S1. We first calculated the surface energy (γ_surface_) of experimentally detected surfaces (Figure S13) as summarized in Table S2 using the following equation:
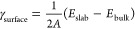
1where *A* represents the surface area of the slab; the terms *E*_slab_ and *E*_bulk_ represent the total energies of the slab and the corresponding bulk which contains the same number of atoms as in the slab. Second, the most stable surfaces were used to create the dual-phase structures for all subsequent calculations. Here, Sn(101), Bi(012), Mg_2_Sn(220), and Mg_3_Bi_2_(012) are the most stable surfaces (see Table S2). Accordingly, the dual-phase surfaces with Mg_3_Bi_2_(012)//Sn(101) and Mg_3_Bi_2_(012)//Mg_2_Sn(220) interfaces were constructed by placing the two surface slab models adjacent to each other with an initial interfacial distance of 3 Å. To minimize the lattice mismatch of these dual-phase surfaces, the Sn(101) 2 × 2 supercell, the Mg_2_Sn(220) 2 × 1 supercell, and the Mg_3_Bi_2_(012) 3 × 1 supercell were constructed to obtain more realistic models for the dual-phase interfaces Mg_3_Bi_2_(012)//Sn(101) and Mg_3_Bi_2_(012)//Mg_2_Sn(220) (see Table S3). These slabs were built with eight atomic layers for the Sn(101) surface models and five atomic layers for Mg_2_Sn(220) and Mg_3_Bi_2_(012) surface models. A vacuum of 15 Å along the *z* direction was added to limit the interaction between the periodic images. To find the most stable interfaces, we considered possible configurations for two dual-phase types and compared their total energies, as illustrated in Figure S14. Note that during initial optimization of these interface models, two surface layers of Bi(012), Mg_3_Bi_2_(012), and Mg_2_Sn(220) and four surface layers for Sn(101) side were fixed in *x* and *y* directions. The most stable interface models for two dual-phase types, as shown in Figure S14, were subsequently optimized without constraints and studied further. The interfacial distances were found to be 0.53 and 1.30 Å for the optimized Mg_3_Bi_2_(012)//Sn(101) and Mg_3_Bi_2_(012)//Mg_2_Sn(220) interfaces, respectively (see Figure S15), where the cell length changes of the pristine surfaces to form the dual-phase structures are reported in Table S3. For all geometry optimizations, the integration of the Brillouin zone using the Monkhorst–Pack scheme^46^ was employed with the moderate k-point density of 5 points per Å as summarized in Table S4.

## Results and Discussion

**Figure 1:**
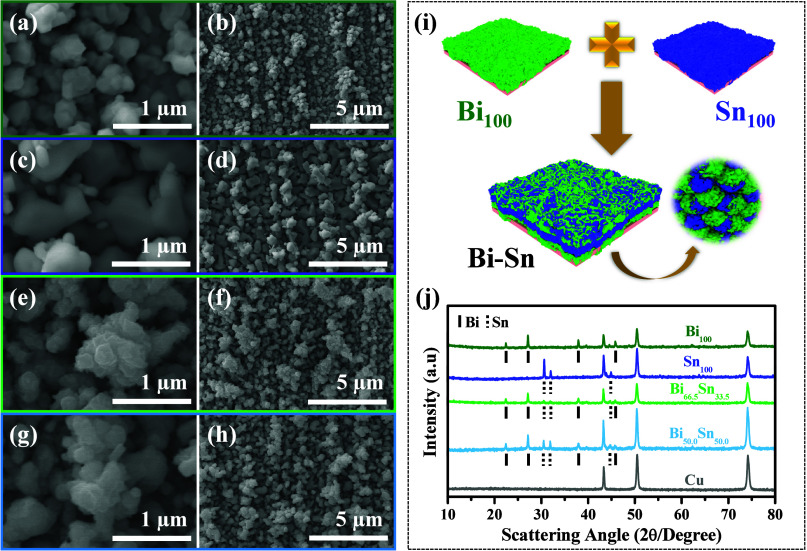
Low and high magnification SEM images of (a, b) Bi_100_, (c, d) Sn_100_, (e, f) Bi_66.5_Sn_33.5_, and (g, h) Bi_50_Sn_50_ anodes. (i) Schematic showing the preparation of Bi–Sn dual-phase anodes and (j) XRD profiles of the anodes.

Thermal evaporation (Figure S1) was used to generate binder-free electrodes with varying compositions of Bi_*x*_Sn_*y*_. SEM of the Bi_100_ electrode (Figure 1a,b) showed a uniform deposition of Bi nanoparticles with an average particle size of 150 nm (Figure S2a). In comparison, the Sn_100_ electrode (Figure 1c,d) had a larger average particle size of 800 nm (Figure S2b). SEM images of Bi_66.5_Sn_33.5_ (Figure 1e,f) and Bi_50_Sn_50_ (Figure 1g,h) electrodes showed that Bi was deposited onto larger Sn particles. The elemental composition of the Bi_66.5_Sn_33.5_ dual-phase electrode was examined by EDS mapping (Figure S3). Furthermore, the electrodes were characterized using XRD (Figure 1j) and showed that thermally evaporated Bi–Sn dual-phase electrodes consisted of rhombohedral Bi (JCPDS no. 04-7365) and tetragonal Sn (JCPDS no. 01-0926) phases. No peaks or phases were detected, corresponding to Bi–Sn alloys.

**Figure 2:**
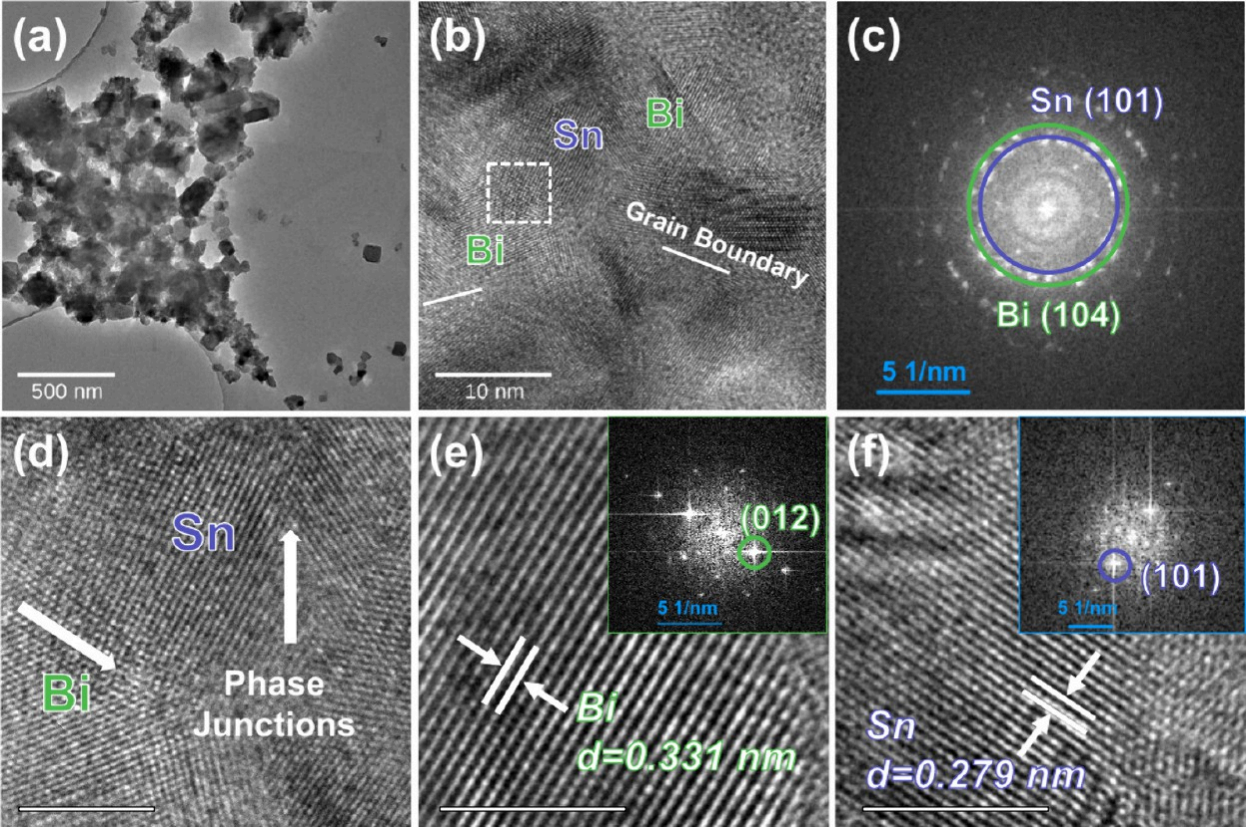
TEM images of Bi_66.5_Sn_33.5_ dual-phase anode: (a) low magnification TEM image, (b) HRTEM image showing the existence of both Bi and Sn phases with clear grain boundaries, (c) FFT corresponding to images (b) and (d), HRTEM image showing Bi–Sn phase junctions, and (e, f) enlarged HRTEM image of Bi and Sn regions with inset showing FFTs (scale bars are 5 nm).

TEM of the Bi_66.5_Sn_33.5_ dual-phase material (Figure 2a) showed distinct regions of Bi and Sn. The HRTEM image (Figure 2b) demonstrates clear lattice fringes of Bi and Sn with lattice spacings of 0.236 and 0.277 nm, corresponding to Bi (104) and Sn (101) planes, respectively. The coexistence of Bi and Sn phases was further confirmed by corresponding FFT rings and integrated Fourier transform as shown in Figures 2c and S4, respectively. Several grain boundaries can be seen in Figure 2b, which can act as microchannels for guest ion transportation. Phase junctions between Bi and Sn were also observed, as shown in Figure 2d. Figure 2e and Figure 2f show enlarged HRTEM images with clear lattice fringes and corresponding FFTs (insets) related to Bi (012) and Sn (101) planes, respectively.

**Figure 3:**
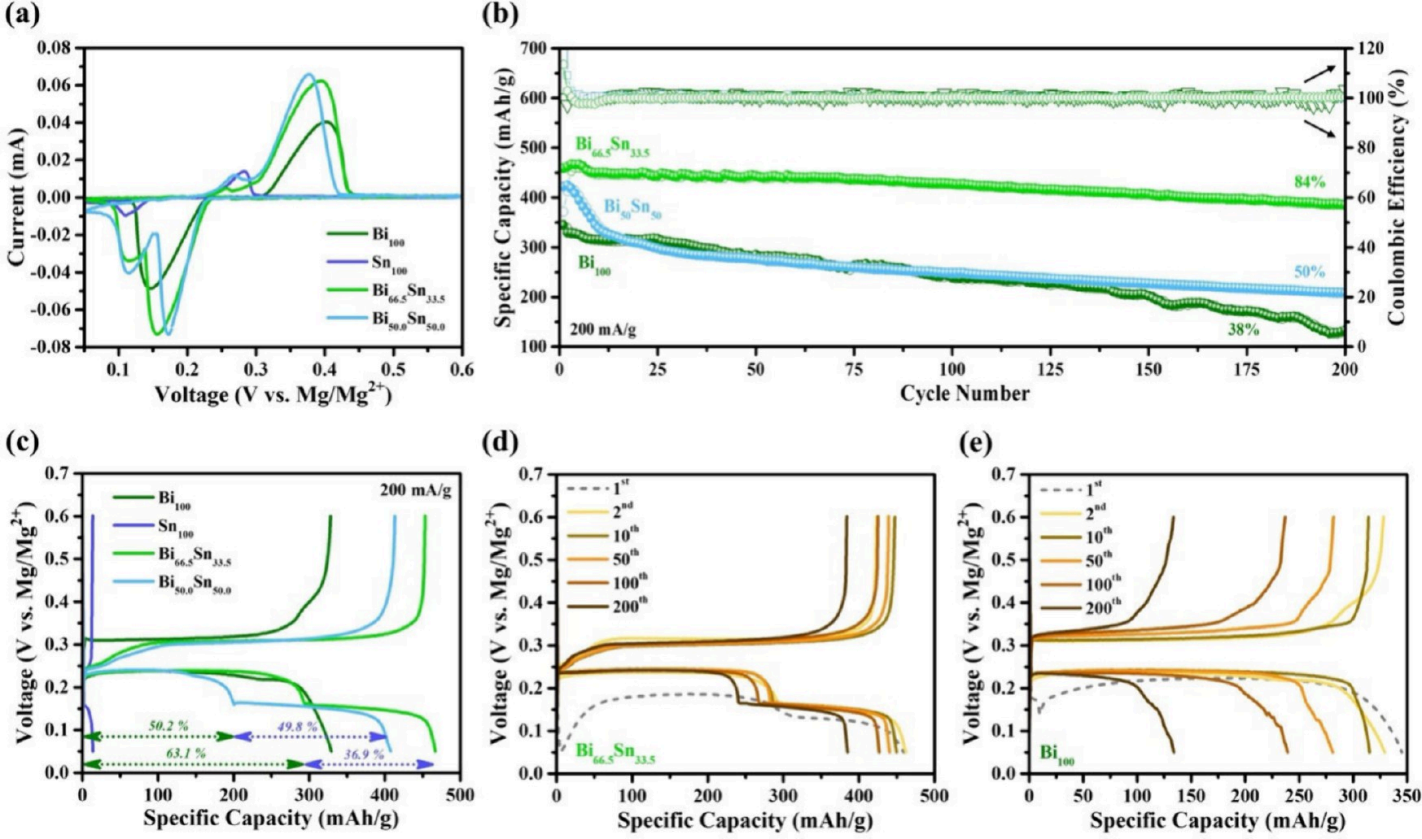
Electrochemical characterization of Bi_100_, Sn_100_, Bi_66.5_Sn_33.5_, and Bi_50_Sn_50_ electrodes: (a) CV curves at 0.1 mV·s^–1^, (b) cycle life of different electrodes, (c) galvanostatic charge–discharge curves, (d) voltage profiles for Bi_66.5_Sn_33.5_ electrode, and (e) voltage profiles of Bi_100_ electrode.

Figure 3a shows the CV profiles of the different electrodes at 0.1 mV s^–1^ between 0.05 and 0.6 V vs Mg^2+^/Mg. The single-phase Sn_100_ electrode shows peaks at 0.11/0.28 V vs Mg^2+^/Mg, corresponding to magnesiation and demagnesiation of Sn, with very low peak intensities confirming the poor reactivity.^19^ The single-phase Bi_100_ electrode exhibited a peak at 0.14 V vs Mg^2+^/Mg for magnesiation and one at 0.40 V vs Mg^2+^/Mg corresponding to demagnesiation of Bi with significantly larger peak intensities, which confirms its ability to reversibly alloy/dealloy with Mg^2+^.^16^ Interestingly, the Bi_66.5_Sn_33.5_ and Bi_50_Sn_50_ electrodes exhibited coupled pairs of redox peaks (indicating two-step reaction mechanisms for magnesiation and demagnesiation), indicating the activity of the Bi and Sn. Similar to the Bi_100_ electrode, the Bi_66.5_Sn_33.5_ electrode exhibited a peak at 0.16 V vs Mg^2+^/Mg corresponding to magnesiation of Bi and one at 0.39 V vs Mg^2+^/Mg for demagnesiation of Bi. The additional cathodic peak at 0.11 V vs Mg^2+^/Mg shows the magnesiation of Sn and the anodic peak at 0.26 V vs Mg^2+^/Mg shows demagnesiation of Sn, which verifies the dual alloying nature of the anode. The CV profiles of the Bi_66.5_Sn_33.5_ electrode showed no significant change in peak intensities for 20 cycles, indicating the stability of this composition (Figure S5a). During galvanostatic testing, the Bi_100_ electrode exhibited fast decay with a capacity retention of only 38% after 200 cycles (Figure 3b). The Sn_100_ electrode revealed a very low capacity but with no obvious decay (Figure S6b). Bi_66.5_Sn_33.5_ delivered significantly improved capacity retention of up to 84% after 200 cycles with an average Coulombic efficiency (CE) of more than 99.5%. Unlike previous reports, the CE of the electrodes achieved its maximum value within a few cycles, which confirms the absence of significant side reactions. The charge–discharge hysteresis of Bi_66.5_Sn_33.5_ was much smaller than that of the Bi_100_ electrode, indicating the decreased polarization effect and stable cycling (Figure 3d,f). Plateaus observed in the voltage profiles correspond to alloying (to form Mg_3_Bi_2_ and Mg_2_Sn) and their dealloying processes.

The voltage profiles of single-phase (Bi_100_, Sn_100_) and dual-element (Bi_66.5_Sn_33.5_ and Bi_50_Sn_50_) electrodes were compared at the current density of 200 mA g^–1^ (Figure 3c), which showed that all materials exhibited voltage plateaus corresponding to magnesiation and demagnesiation of Bi and/or Sn. It is worth noting that because Bi and Sn expand (and contract) at different potentials during the magnesiation (demagnesiation), not all of the active material is expanding at the same time during cycling. The experimental results revealed that 50 wt % of Bi was sufficient to enable activation of the 50 wt % of Sn, but capacity dropped in following cycles. The compositional “sweet-spot” appears to be where the redox reactions are dominated by Mg_3_Bi_2_ alloy formation with Mg_2_Sn alloy formation contributing a smaller fraction of the capacity. Increasing the amount of Sn in the anode leads to a greater % of the initial capacity being contributed from Sn (see Figure 3c); however, this portion of the capacity (i.e., the part from the Sn in this Sn rich anode) is less stable over repeated cycles than in the Bi_66.5_Sn_33.5_ case (see Figure 3d for illustration of this stability). Therefore, in the long term cycling, we see an initial drop off in the capacity for the Bi_50_Sn_50_ anode as the portion of the capacity from the Sn decreases. The Sn_100_ electrode exhibited a very low specific capacity (13 mAh g^–1^), whereas the Bi_100_ electrode exhibited a specific capacity of 328 mAh g^–1^. On the other hand, Bi_66.5_Sn_33.5_ and Bi_50_Sn_50_ electrodes exhibited improved specific capacity values (467 and 407 mAh g^–1^, respectively). The voltage plateaus at about 0.24 and 0.16 V correspond to the magnesiation of Bi and Sn, respectively, during the discharge process. For the Bi_50_Sn_50_ electrode, ∼50.2% and ∼49.8% of specific capacities were coming from Bi and Sn, respectively. When the Sn content was reduced to 33.5 wt %, the capacity contribution from Sn was also reduced to ∼36.9%, which is consistent with the sample composition. Postcycling SEM analysis of all electrodes revealed a transformation of the active material into a structure with reduced particle size as shown in Figure S7. Unlike the Bi_66.5_Sn_33.5_ and Bi_50_Sn_50_ electrodes, which were robust, the Bi_100_ was completely peeled off from the substrate due to huge volume variation caused by repeated alloying/dealloying, thus explaining the fast capacity decay. This demonstrates that the Sn acts as a buffer in the dual electrodes, absorbing some of the mechanical stress caused by the volume changes of Bi during cycling. This reduces the overall strain on the electrode, minimizing the structural degradation that occurs in pure Bi. The interaction between Bi and Sn in the composite creates a more stable matrix. This dual-phase structure distributes the volume changes more evenly across the electrode, preventing localized stress and maintaining the structural integrity of the anode.

**Figure 4:**
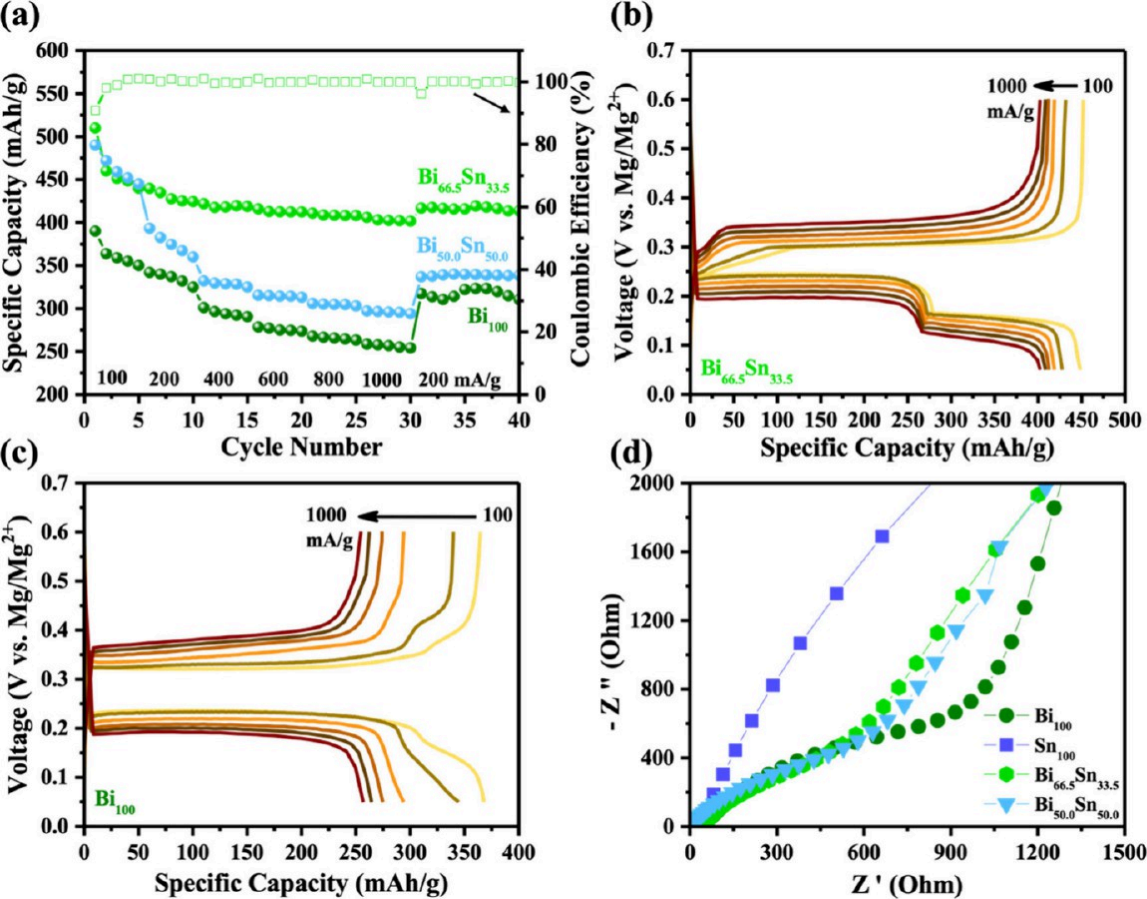
(a) Rate capabilities of Bi_100_, Bi_66.5_Sn_33.5_, and Bi_50_Sn_50_ electrodes for Mg discharging, corresponding voltage profiles of Bi_66.5_Sn_33.5_ (b) and Bi_100_ (c) electrodes, and (d) Nyquist plots measured after 2nd cycle.

Rate capability testing revealed that Sn_100_ exhibited very poor reversible capacities due to sluggish Mg^2+^ ion transportation in the Sn lattice (Figure S6a),^19^ whereas Bi_100_ electrode exhibited reversible capacities of 361, 336, 294, 273, 265, and 253 mAh g^–1^ at the current densities of 100, 200, 400, 600, 800, and 1000 mA g^–1^, respectively (Figure 4a). Bi_66.5_Sn_33.5_ exhibited excellent rate capability with high reversible capacities of 462, 427, 419, 412, 409, and 403 mAh g^–1^ at the current densities of 100, 200, 400, 600, 800, and 1000 mA g^–1^, respectively, and CE of more than 99.5%. Compared to Bi_66.5_Sn_33.5_, Bi_50_Sn_50_ exhibited faster capacity decay due to the presence of more Sn, which exhibits faster capacity decay than Bi at high current rates as shown in Figure S6a. The voltage profiles (Figure 4b,c) revealed obvious plateaus for Bi_66.5_Sn_33.5_ even at higher current, which suggests that the dual element electrode effectively improves Mg^2+^ ion transportation. The performance shows competitive capacity values compared to previously reported dual electrodes (Sn–Sb,^27^ Bi–In,^28^ Bi–Pb,^29^ Ga–Sn,^30^ and Bi–Sn.^32^ Compared with the previously reported Bi–Sn anode,^32^ our Bi–Sn dual-phase anodes showed higher capacities and enhanced cycle life, albeit our electrodes have lower mass loading. Future studies on dual electrodes should focus on delivering much higher areal loadings (>10 mg/cm^2^) for practical areal capacities. Electrochemical impedance spectroscopy (EIS) was used to gain an in-depth understanding of the interfacial charge transfer and Mg^2+^ ion diffusion process as shown in Figures 4e and S6d–f. The Sn_100_ electrode exhibited very high *R*_ct_ (270 ×10^3^ Ω), whereas the Bi_100_ electrode exhibited much lower *R*_ct_ (1076 Ω) after second cycle. On the contrary, the Bi_66.5_Sn_33.5_ and Bi_50_Sn_50_ electrodes exhibited reduced *R*_ct_ values, i.e., 581 and 642 Ω, respectively, attributed to faster charge transfer and Mg^2+^ ion diffusion.

**Figure 5:**
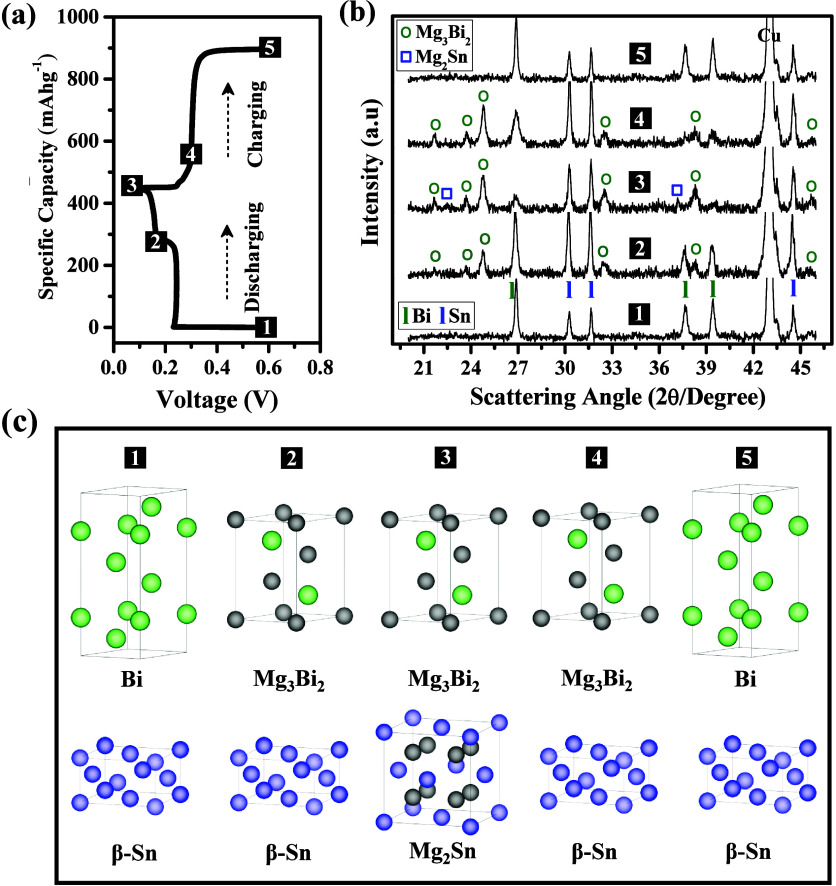
(a) Galvanostatic charging–discharging curves of Bi_66.5_Sn_33.5_ dual-phase electrode, (b) ex situ XRD profiles of electrodes at different charge–discharge states (1–5) corresponding to (a), and (c) scheme representing the structural evolutions of the Bi–Sn dual-phase electrode at various charge–discharge states corresponding to XRD profiles in (b).

XRD profiles of dual-phase Bi_66.5_Sn_33.**5**_ electrodes at distinct stages of the cycling (as marked in Figure 5a) of the second cycle are shown in Figure 5b. At point 1 (∼0.6 V), the demagnesiated electrode exhibits peaks corresponding to rhombohedral Bi (JCPDS no. 04-7365) and tetragonal Sn (JCPDS no.01-0926) phases. When the electrode was discharged to point 2 (from ∼0.22 V to ∼0.15 V), additional peaks corresponding to the formation of hexagonal Mg_3_Bi_2_ (JCPDS no. 03-6859) were detected, suggesting that the first plateau belongs to the alloying of Mg with Bi. With further discharge to point 3 (∼0 V), peaks linked to formation of cubic Mg_2_Sn (JCPDS no. 08-3224) were also traced along with the remaining Bi and Sn peaks with reduced intensities. At this stage, the electrode was fully magnesiated, indicating that the second discharge plateau belongs to the alloying of Mg with Sn to form Mg_2_Sn. It is important to mention that the electrode needs a few cycles to completely transform the remaining crystalline Bi or Sn into their fully alloyed forms. When the electrode was charged to point 4 (∼0.29 V), the Mg_2_Sn was dealloyed to form crystalline Sn, while fully charging (demagnesiated) to point 5 (∼0.6 V) showed the disappearance of Mg_3_Bi_2_. The structural evolution of the dual-phase Bi–Sn electrode at the discussed charge and discharge states (1–5) is summarized in Figure 5c, which shows the formation and deformation of Mg_3_Bi_2_ and Mg_2_Sn phases into Bi and Sn, respectively. In contrast, XRD of the Bi_100_ electrode at different stages (Figure S8) showed the formation of hexagonal Mg_3_Bi_2_ (JCPDS no. 03-6859) upon complete magnesiation. The other peaks with reduced intensities are linked to remnant Bi. Upon demagnesiation, the Mg_3_Bi_2_ transformed back into crystalline Bi. TEM and STEM analyses confirmed the presence of Mg_3_Bi_2_. The STEM images and corresponding EDX maps of the magnesiated electrode revealed that Mg is distributed equally along with Bi in the sample, indicating the formation of Mg_3_Bi_2_ (Figure S8). HRTEM analysis of the demagnesiated electrode indicates that the Mg_3_Bi_2_ alloy fully reverts to the crystalline Bi phase, which is consistent with the ex situ XRD profile.

**Figure 6:**
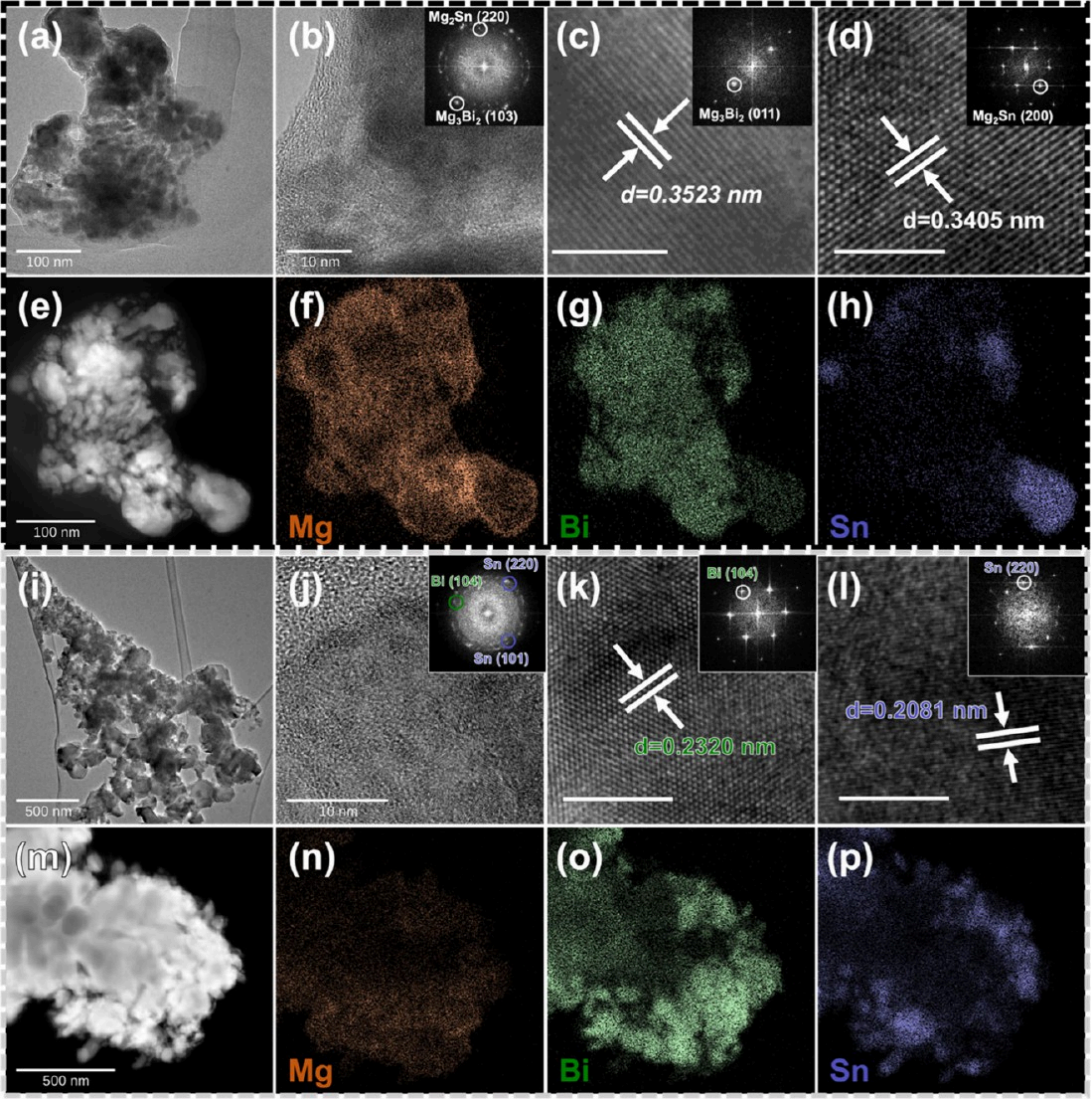
(a) TEM image of magnesiated electrode. (b) HRTEM image showing formation of Mg_3_Bi_2_ and Mg_2_Sn phases with inset showing corresponding FFT. (c, d) Enlarged HRTEM images of Mg**_3_**Bi**_2_** and Mg_2_Sn phases with insets showing corresponding FFTs. (e–h) STEM images of electrode in magnesiated states showing elemental maps. (i) TEM image of demagnesiated electrode. (j) HRTEM image showing Bi and Sn phases with inset showing corresponding FFTs. (k, l) Enlarged HRTEM images of Bi and Sn phases with insets showing their corresponding FFTs and (m–p) STEM images of electrode in demagnesiated states showing the elemental maps. Scale bars in (c), (d), (k), and (l) are 5 nm.

Ex situ TEM and STEM were used to analyze Bi_66.5_Sn_33.5_ in the magnesiated (Figure 6a–h) and demagnesiated (Figure 6i–p) states. The FFT of the HRTEM image taken of the magnesiated electrode exhibits rings corresponding to the (103) plane of Mg_3_Bi_2_ and the (220) plane of Mg_2_Sn (Figure 6b). The enlarged HRTEM images (Figure 6c,d) exhibit clear lattice fringes with lattice spacings of 0.3523 and 0.3405 nm corresponding to the Mg_3_Bi_2_(011) and Mg_2_Sn(200) planes, respectively. Furthermore, STEM images of the magnesiated electrode revealed that Mg is dispersed evenly within the sample, which indicates the alloying reactions of Mg (with Bi and Sn) to form Mg_3_Bi_2_ and Mg_2_Sn (Figure 6e–h). The Bi and Sn revealed alternate distributions (i.e., spatial segregation), confirming the presence of interspersed Mg_3_Bi_2_ and Mg_2_Sn phases. The FFT for the demagnesiated electrode showed spots corresponding to the (104) plane of Bi and (220) plane of Sn (Figure 6j). Similarly, the enlarged HRTEM images (Figure 6k,l) exhibit lattice fringes with lattice spacings of 0.232 and 0.208 nm corresponding to Bi(104) and Sn(220) planes, respectively. Also, the STEM images (Figure 6m–p) of a demagnesiated electrode revealed that the intensity of the Mg map was significantly reduced, indicating the dealloying of Mg_3_Bi_2_ and Mg_2_Sn to form Bi and Sn phase as confirmed by ex situ XRD data discussed above. The integrated Fourier transforms of HRTEM images (Figure 6b,d,c,j,k,l) are shown in Figure S10, which also validates the existence of Bi, Sn, Mg_3_Bi_2_, and Mg_2_Sn phases in the magnesiated and demagnesiated states.

To corroborate the experimental results, the mechanism of Mg introduction in the Bi–Sn dual-phase was investigated using DFT calculations (see Computational Details section and additional justification for model parameters in Supporting Information). We first optimized the unit cells of β-Sn, Mg_2_Sn, Bi, and Mg_3_Bi_2_ (Figure S11) and calculated their lattice parameters (Table 1). The DFT-optimized lattice parameters are close to the experimentally measured values.

**Figure 7:**
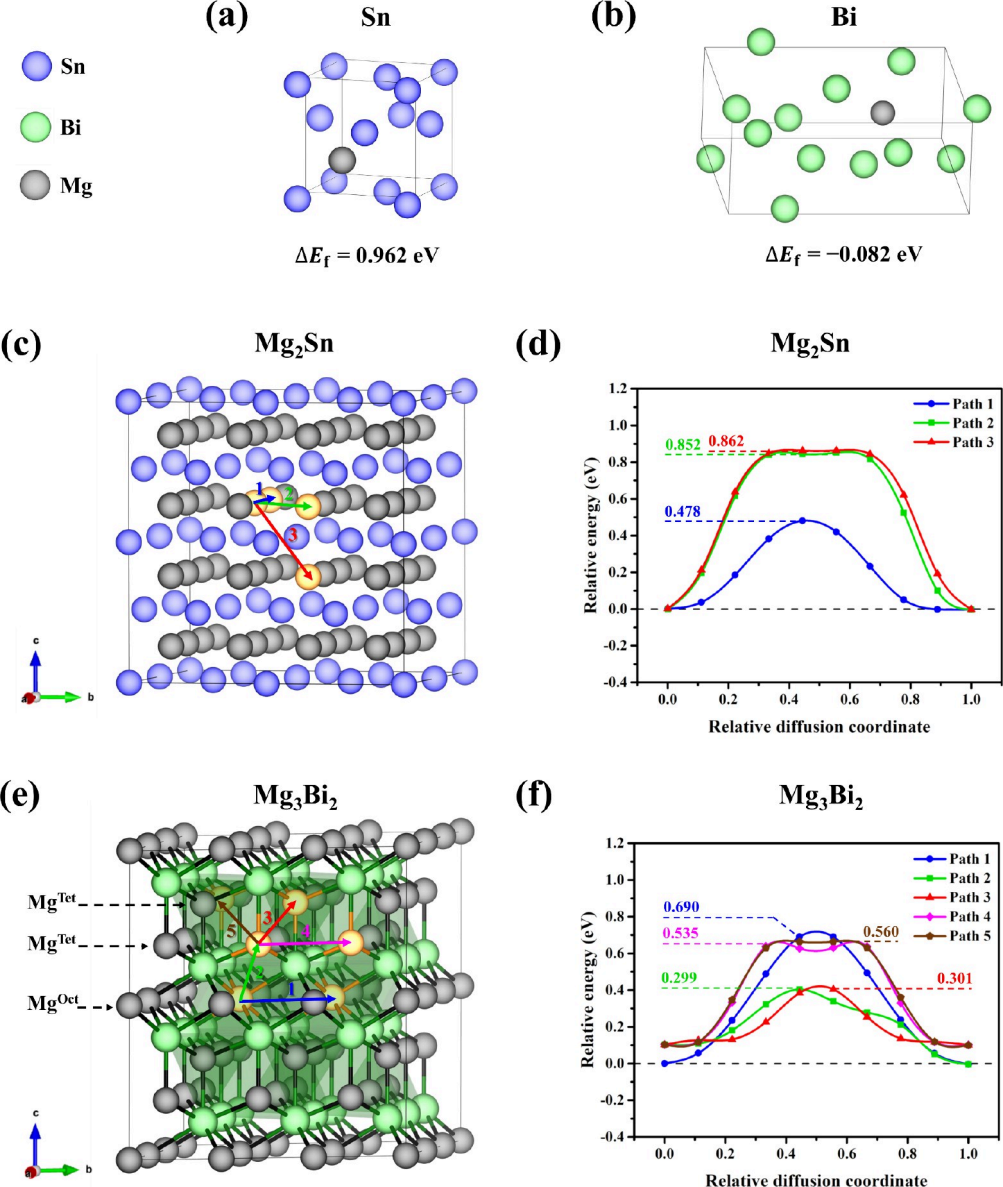
Interstitial Mg insertion in relevant (a) Sn lattice and (b) Bi lattice with their Mg insertion energy (Δ*E*_f_, eV). Schematic illustration of Mg diffusion pathways and potential energy profiles of all considered migration paths for Mg_2_Sn (c, d) and Mg_3_Bi_2_ involving tetrahedrally and octahedrally coordinated Mg atoms, Mg^Tet^ and Mg^Oct^ (e, f), with the calculated barriers in eV. The relative energies are referenced to the energy of the most stable Mg_2_Sn (d) and Mg_3_Bi_2_ (f) containing a Mg vacancy.

Next, the behavior of Mg ion insertion was investigated by calculating the insertion energy (Δ*E*_f_) of Mg in pristine Sn and Bi:
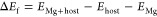
2where *E*_Mg+host_, *E*_host_, and *E*_Mg_ represent the total energy of an inserted Mg atom in the host material, the total energy of the host material, and the total energy of Mg atom, respectively. Mg was inserted in β-Sn and in four different ways for Bi (Figure S16). The most stable Mg insertion energies were 0.962 eV and −0.082 eV for β-Sn and Bi unit cells, respectively. The volume expansion upon Mg insertion is 39% and 11% for Sn and Bi, respectively (insertion configurations are illustrated in Figure 7a,b). Additionally, Mg insertion in the 2 × 2 × 4 Sn and 3 × 3 × 1 Bi supercells was also considered to explore the effect of the model system size. The most stable Mg insertion energies were 0.544 and 0.301 eV with a volume expansion of 2% and 1% for Sn and Bi supercells, respectively. This indicates that the system size definitely has a significant impact on the Mg insertion energy and structural relaxation. Nevertheless, the calculations of unit cells and supercells provide similar findings in that it is easier to insert Mg into Bi compared to Sn, with less lattice expansion. Thus, Mg insertion starts in the Bi phase, corroborating the formation of the fully magnesiated Bi phase (Bi_2_Mg_3_) before Mg_2_Sn, observed experimentally. Interestingly, the formation of both bulk magnesiated phases is exotherm by −0.324 eV/Mg for a Mg_2_Sn unit cell and −0.377 eV/Mg for a Bi_2_Mg_3_ unit cell (−0.320 eV/Mg for 2 × 2 × 2 Mg_2_Sn supercell and −0.376 eV/Mg for 3 × 3 × 2 Bi_2_Mg_3_ supercell).

To evaluate the kinetics of initial demagnesiation, we further determined Mg diffusion barriers in fully magnesiated phases of Sn and Bi, i.e., Mg_2_Sn and Mg_3_Bi_2_ through a Mg-vacancy diffusion mechanism. First, a single Mg vacancy was created, followed by examination of various migration pathways (Figure 7c) to unravel their corresponding diffusion barriers (Figure 7d). A Mg ion can move to the first nearest neighbor (3.41 Å) in the same Mg layer according to path 1, by overcoming a barrier of 0.479 eV, whereas Mg has to overcome a high barrier of 0.852 eV to migrate to the second nearest neighbor (4.82 Å) into a vacancy in case of path 2. For the diffusion to an adjacent Mg layer (5.91 Å), path 3, the calculated energy barrier is similar to path 2 (0.862 eV); in the latter case Mg needs to pass first through an interstitial void in between two Sn atoms in a Sn layer. Furthermore, in a similar fashion, the Mg diffusion behavior was explored in Mg_3_Bi_2_, determining the energy profiles for five possible vacancy diffusion pathways (Figure 7e,f). In Mg_3_Bi_2_, there are octahedrally (Oct) and tetrahedrally (Tet) coordinated Mg atoms, which result in one Mg^Oct^ layer and two Mg^Tet^ layers in between Bi layers (in the *c* direction). When Mg^Oct^ moves to a nearest neighbor vacancy in the same Mg layer (4.71 Å), there is a large energy barrier of 0.690 eV (path 1). Path 2 represents an Mg^Oct^ migrating to a neighboring Mg^Tet^ layer (3.89 Å). As the position of Mg^Tet^ is more stable, it is easier for Mg to move from the Oct site to Tet site with an energy barrier of 0.299 eV. For a Mg^Tet^ moving to three neighboring Tet sites, i.e., path 3 (3.32 Å), path 4 (4.71 Å), and path 5 (5.76 Å), the corresponding energy barriers are 0.301, 0.535, and 0.560 eV, respectively. It is obvious that the energy barriers of Mg migration in Mg_3_Bi_2_ (in the range of 0.299–0.690 eV) are lower than those of in Mg_2_Sn (0.479–0.862 eV), in agreement with a previous theoretical study.^33^ The results can be used to rationalize why Bi can be a better anode material for Mg-ion batteries compared to Sn.

To get insights into the role of Bi in enhancing the electrochemical reactivity of Sn within the dual-phase Bi–Sn, we explored the thermodynamic stability of Bi–Sn alloys where a Sn atom was replaced by a Bi atom in the Sn system and vice versa (Figure S16). Then, the doping energy (Δ*E*_doping_) of Bi in Sn and Sn in Bi was calculated as follows:
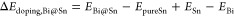
3a
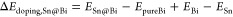
3bwhere Δ*E*_Bi@Sn_ and Δ*E*_Sn@Bi_ represent the total energy of a single Bi atom substituted in a Sn system and a Sn atom substituted in a Bi system, respectively; *E*_pureSn_ and *E*_pureBi_ represent the total energy of pure Sn and Bi, respectively; *E*_Sn_ and *E*_Bi_ represent the energy of an atom in bulk β-Sn or Bi (Table 1), respectively. The calculated doping energies of Bi in Sn and Sn in Bi were found to be 0.127 and 0.057 eV, respectively. This is an indication that the initial Bi–Sn alloy formation from Bi or from Sn is endothermic, therefore confirming separate Bi and Sn phases. This renders the initial mixing of Bi and Sn energetically unfavorable and thus seems to confirm the experimentally observed segregation between Bi and Sn during synthesis, as characterized using XRD where the peaks corresponding to Bi–Sn alloys were not detected (Figure 1j).

**Figure 8:**
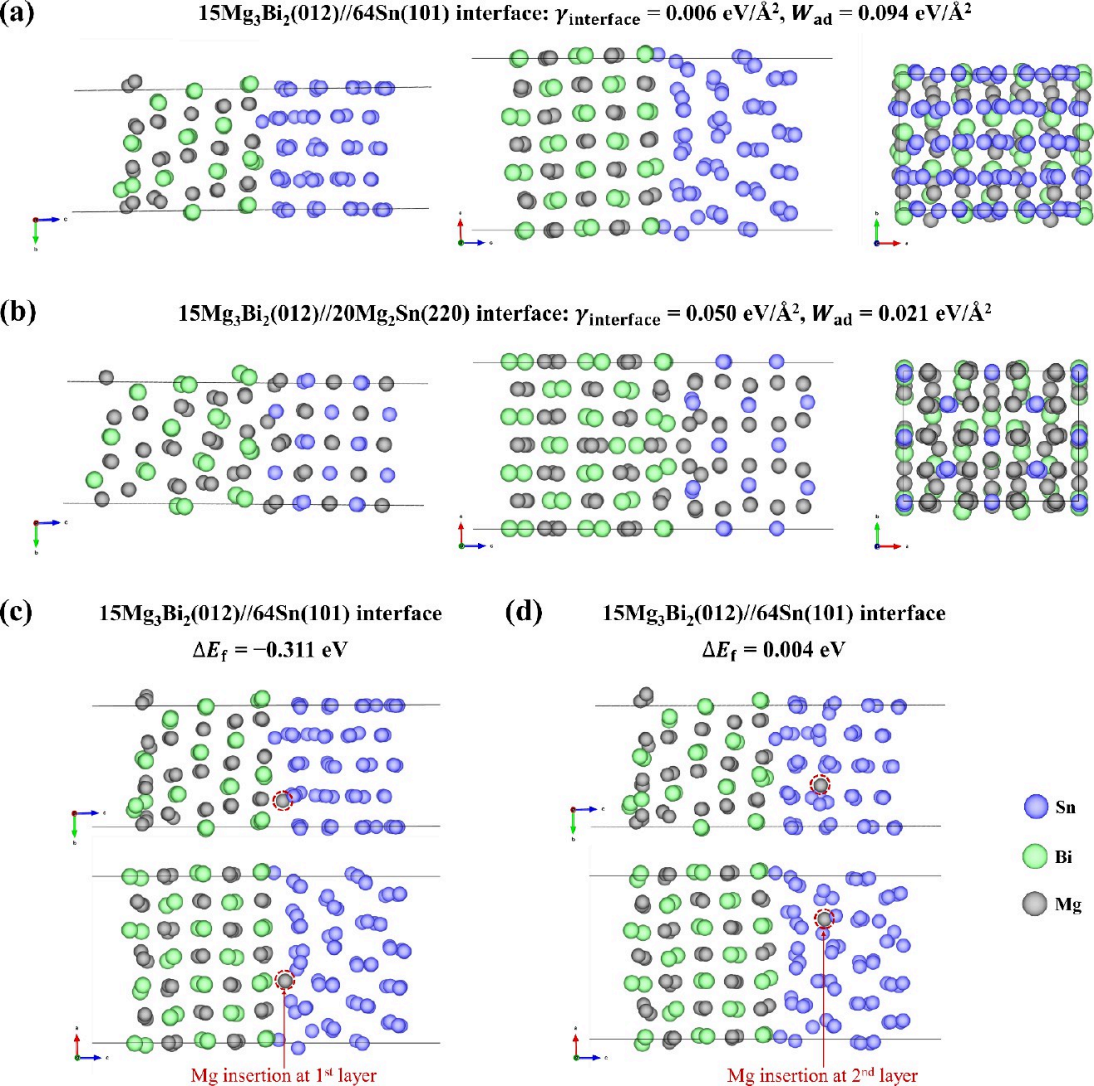
Dual-phase structures of (a) Mg_3_Bi_2_(012)//Sn(101) and (b) Mg_3_Bi_2_(012)//Mg_2_Sn(220) interfaces in different orientations with their interface energy (γ_interface_, eV/Å^2^) and work of adhesion (*W*_ad_, eV/Å^2^). Mg insertion at the (c) first and (d) second layer of Sn in the Mg_3_Bi_2_(012)//Sn(101) interface with their defect formation energy (Δ*E*_f_, eV). The unit cell box lines of the systems are indicated with fine black lines.

Experimental observations point to Mg alloying with Bi prior to alloying with Sn during the discharging process of the dual-phase Bi–Sn electrode. We considered the thermodynamics of interface formation of two dual-phase models, i.e., Mg_3_Bi_2_(012)//Sn(101) and Mg_3_Bi_2_(012)//Mg_2_Sn(220) as illustrated in Figure 8a,b, respectively. The most stable interface structures were achieved as discussed in the Computational Details section. To describe the energetics of the interaction between two materials to form the dual-phase or interface structure, the interface energy (γ_interface_) for X//Y was calculated using the following equation:^47^
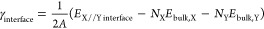
4where *A* represents the surface area of the interface system; and *E*_X//Yinterface_, *E*_bullk,X_, and *E*_bullk,Y_ are the total energies of the interface system, bulk of material X, and bulk of material Y, respectively; *N*_*i*_ denotes the number of formula units of *i* in the interface system. The interface energies were calculated to be 0.006 eV/Å^2^ and 0.050 eV/Å^2^ for Mg_3_Bi_2_(012)//Sn(101) and Mg_3_Bi_2_(012)//Mg_2_Sn(220), respectively. This indicates that low energies are required to create the interfaces from their corresponding bulk. Because it is also easier to insert Mg into Bi than into Sn (vide supra), we assumed that interfaces such as Mg_3_Bi_2_(012)//Sn(101) can exist during the charging and discharging processes. The lower surface energy of the Bi_2_Mg_3_(012)//Sn(101) interface model compared to the Mg_3_Bi_2_(012)//Mg_2_Sn(220) model (Figure S14) seems to confirm the sequential magnesiation. Moreover, the low surface energy observed for the Mg_3_Bi_2_(012)//Sn(101) interface (0.006 eV/Å^2^) shows the clear driving force to form the magnesiated Bi phase (Mg_3_Bi_2_(012)) near the Sn phase; remarkably lower than those of exposed Sn(101) (0.018 eV/Å^2^, Table S2) and Mg_3_Bi_2_(012) (0.026 eV/Å^2^, Table S2). Based on these surface energies, it is more likely to insert Mg into Bi in the presence of Sn than without Sn. On the other hand, the energy required to form the Mg_3_Bi_2_(012)//Mg_2_Sn(220) interface (0.050 eV/Å^2^) is higher than those of the individual Mg_2_Sn(220) (0.027 eV/Å^2^, Table S2) and Mg_3_Bi_2_(012) (0.026 eV/Å^2^, Table S2). This indicates that the Mg_3_Bi_2_(012)//Mg_2_Sn(220) interface has a larger tendency to break upon complete magnesiation of Sn. However, spontaneous demagnetisation in the interface region can also happen, given that the formation of some Mg vacancies is exotherm (Table S5, Figure S17).

Another thermodynamic property of interest is the interface bonding strength, evaluated by the interface work of adhesion or interface binding energy. The work of adhesion (*W*_ad_) is defined as the reversible work required per unit area to separate an interface into two free surfaces, expressed as follows:^48,49^
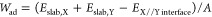
5in which *A* represents the surface area of the interface system; the terms *E*_slab,X_, *E*_slab,Y_, and *E*_X//Yinterface_ represent the total energies of the surface slab of material X, surface slab of material Y, and the interface system, respectively. The positive *W*_ad_ implies that the two surfaces will bond, and the higher the value of *W*_ad_, the stronger is the interaction between the two surfaces upon formation of interface. The calculated *W*_ad_ values of Mg_3_Bi_2_(012)//Sn(101) and Mg_3_Bi_2_(012)//Mg_2_Sn(220) interfaces are 0.094 eV/Å^2^ and 0.021 eV/Å^2^, respectively.

Next, the Mg insertion can be studied for a Mg_3_Bi_2_(012)//Sn(101) interface, i.e., the insertion of a Mg atom in the Sn phase near the Mg_3_Bi_2_ phase. Here, Mg insertion in the first layer and second layer of Sn with respect to the Mg_3_Bi_2_(012)//Sn(101) interface were considered (Figure 8c and Figure 8d, respectively). The tensile areal strain of 0.51% is caused upon the Mg insertion in the first layer of Sn while the compressive areal strain is found to be 0.53% when Mg is inserted into the second layer of Sn. According to eq 2, the calculated Mg defect formation energies (Δ*E*_f_) are −0.311 and 0.004 eV for Mg insertion at the first and second layer, respectively. These results indicate that Mg insertion in the case of the Bi_2_Mg_3_//Sn interface model happens preferably at the interface, and Mg can be inserted easier into Sn in the neighborhood of a Mg_3_Bi_2_ phase compared with pristine Sn (Δ*E*_f_ = 0.962 eV in a Sn unit cell and Δ*E*_f_ = 0.544 eV in 2 × 2 × 4 Sn supercell). This suggests that the formation of the Mg_3_Bi_2_ phase promotes the Mg insertion in Sn and improves the electrochemical reactivity of Sn. Our results are in good agreement with a previous DFT study that demonstrated the easier insertion of Mg in Sn of Bi–Sn system as benchmarked with pure Sn.^32^ Moreover, it is also very clear that Mg discharging happens more easily at the material interfaces between Mg_3_Bi_2_ and Mg_2_Sn, compared to bulk magnesiated phases (compare Mg vacancy formation energy for Mg_3_Bi_2_(012)//Mg_2_Sn(220) interface models with bulk Mg_3_Bi_2_ and Mg_2_Sn in Table S5). Thus, the lower Mg defect formation energy at the material interfaces is one of the key performance improvements offered by employing dual-phase Bi–Sn electrodes.

## Conclusions

We have successfully demonstrated a straightforward thermal evaporation technique to design binder-free, dual-phase Bi–Sn electrodes for MIBs. The electrodes with interspersed element distribution have high reversible capacities, excellent rate capability, and stable cycling performance when compared with single-phase Bi or Sn. Ex situ analysis revealed that the discharge process of dual-phase Bi–Sn electrodes consists of two steps with consecutive reaction of Mg^2+^ first with Bi and then with Sn to generate Mg_3_Bi_2_ and Mg_2_Sn alloys, respectively, and vice versa to regenerate nanosized Sn and Bi particles, which are more active to Mg^2+^ ions. Using a dual-phase strategy, we have successfully activated Sn by in situ formation of electrochemically active Sn in the presence of Mg_3_Bi_2_. The superior performance of the dual-phase Bi–Sn electrodes was attributed to the increased density of phase and grain boundaries, which act as channels for the fast transportation of guest ions. The multistep phase transition process and formation of dealloyed nanoporous structures not only shortens the diffusion lengths but also accommodates the volume expansions. DFT calculations revealed alloy formation of Bi and Sn is energetically unfavorable, confirming the experimentally observed dealloying of the dual-phase Bi–Sn electrodes. Since the initial insertion of Mg into Bi is less endothermic than for Sn, Mg insertion is likely to occur first in the Bi phase, subsequently forming the fully magnesiated Biphase (Bi_2_Mg_3_) surrounded by Sn prior to the formation of Mg_2_Sn as observed in experiments. Notably, the formation of the bulk magnesiated phases is exothermic for both Mg_2_Sn and Bi_2_Mg_3_ whereas Mg diffusion is less activated in Bi_2_Mg_3_ compared to Mg_2_Sn. Moreover, the calculated surface energies indicate that the formation of the Bi_2_Mg_3_//Sn interface model is more favorable compared to the individual Bi_2_Mg_3_ and Sn surfaces. Dealloying Mg from Mg_3_Bi_2_:Mg_2_Sn systems requires the creation of Mg vacancies and subsequent Mg diffusion. Mg vacancy creation is thermodynamically easier for Mg_2_Sn compared to Mg_3_Bi_2_, while the latter has slightly lower activated Mg-diffusion pathways. However, considering the lower Mg vacancy creation energy at the interfaces of both phases (e.g., Mg_2_Sn(220):Mg_3_Bi_2_(012)), initial Mg discharging will take place there and afterward be kinetically limited by Mg diffusion barriers. The DFT results corroborate the experimentally improved electrochemical performance of the dual-phase Bi–Sn electrodes over pure Sn or pure Bi. The employed synthesis method is economical and easy to scale up for commercial application. The proposed dual-phase concept can potentially boost the electrochemical reactivity of the less active group IV elements (i.e., Ge and Si) with high defect formation energy using IV–Bi dual-phase engineering.

## Supporting Information Available

Figures showing thermal evaporation method, particle sized, elemental mapping, HRTEM and CV results, profiles of parameters, morphology, STEM results, and background to DFT calculations (PDF)
