# Supplementary material for: Enhancing Magnesium-Ion Storage in a Bi–Sn Anode through Dual-Phase Engineering
Source: ACS Appl Mater Interfaces. 2024 Oct 5;16(41):55383–94. doi: 10.1021/acsami.4c11272 (PMC11492170; doi:10.1021/acsami.4c11272)
Supplement: Supplementary file 1 — am4c11272_si_001.pdf [file am4c11272_si_001.pdf]

Supporting Information for

Enhancing Magnesium-Ion Storage in a Bi-Sn Anode through Dual-Phase Engineering

Muhammad Rashad, Apinya Ngoipala, Matthias Vandichel,[#] Hugh Geaney[#]
Department of Chemical Sciences and Bernal Institute, University of Limerick, Limerick V94 T9PX, Ireland

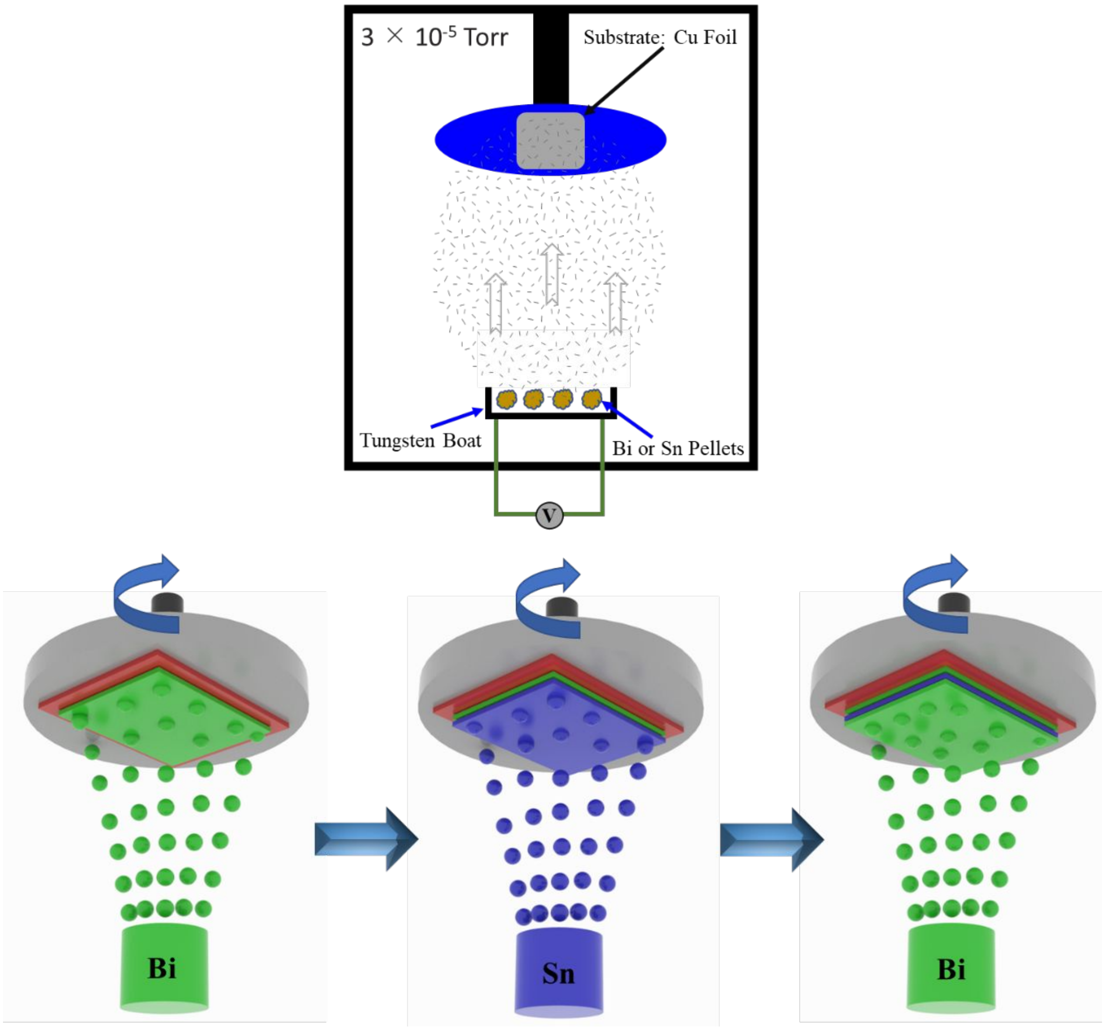

Figure S1 Sketch diagram showing the basic operation principle of the thermal evaporation method to deposit the Bi, Sn, and Bi-Sn onto a substrate.

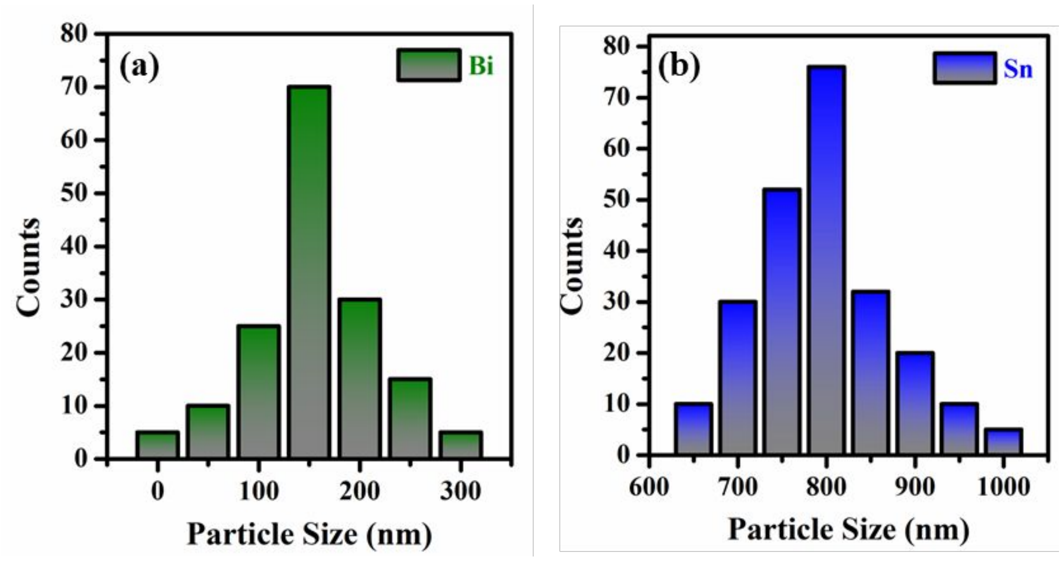

Figure S2 Particle size distribution graphs of (a) Bi, and (b) Sn electrodes.

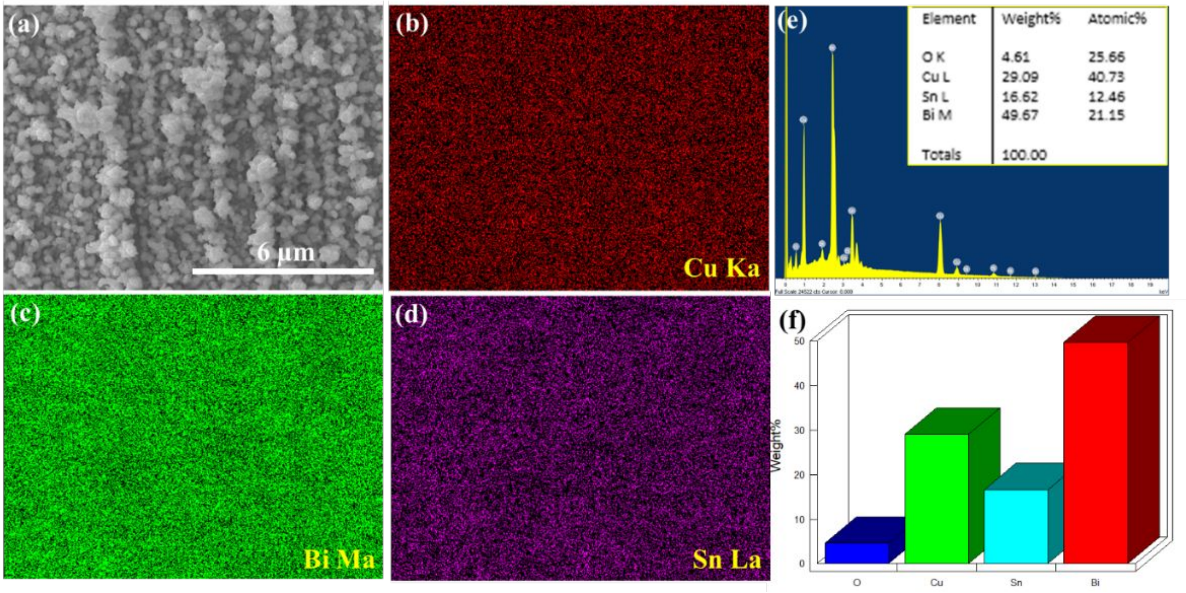

Figure S3 EDS elemental mapping (a-d) of $Bi_{66.5}Sn_{33.5}$ dual-phase electrode and corresponding spectrum (e), and weight percentage analysis of Sn and Bi (f).

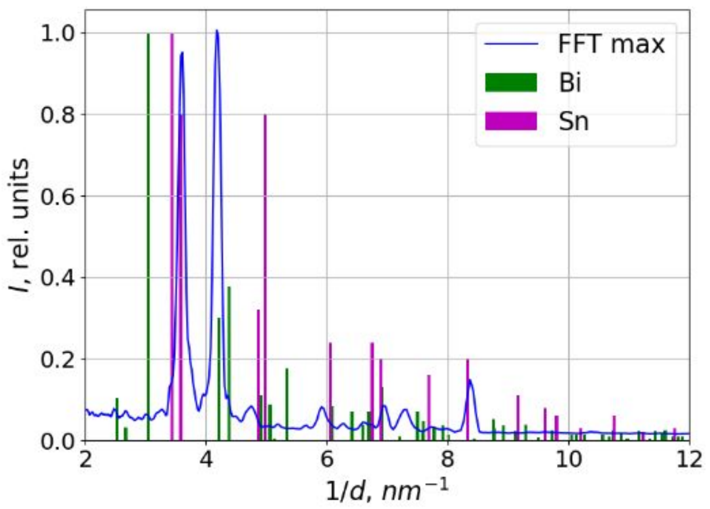

Figure S4 Integrated Fourier-transform of HRTEM image (Figure 2(b)) and bars corresponding to the Bi and Sn phases.

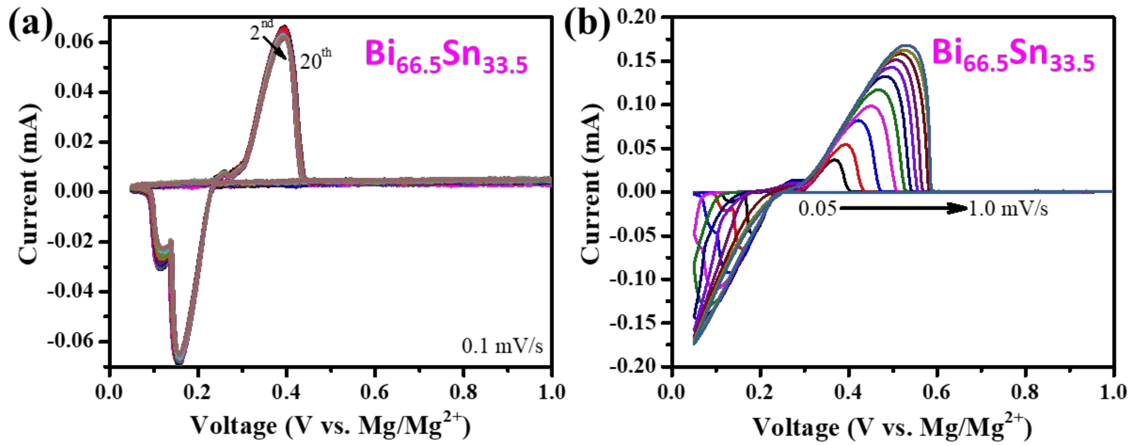

Figure S5 CV profiles of $Bi_{66.5}Sn_{33.5}$ dual-phase electrode at (a) 0.1 mVs^{-1} / 2nd – 20th cycle, and (b) 0.05 – 1.0 mVs^{-1}.

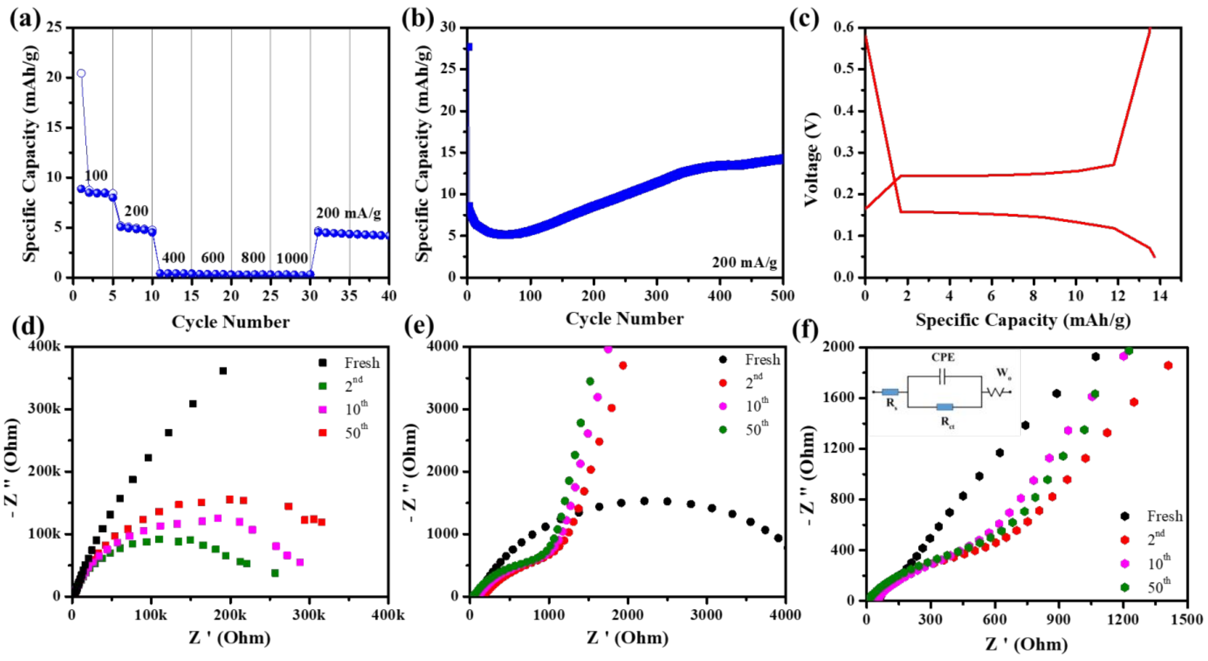

Figure S6 (a) Rate capability, (b) cycle stability, and (c) voltage profile of Sn_{100} electrode, Nyquist plots of (d) Sn_{100}, (e) Bi_{100}, and (f) $Bi_{66.5}Sn_{33.5}$ electrodes after different number of cycles and inset in (f) shows the equivalent circuit, where R_s, R_{ct}, CPE, and W_o represent solution resistance of the cell, charge transfer resistance, capacitance, and Warburg factor, respectively.

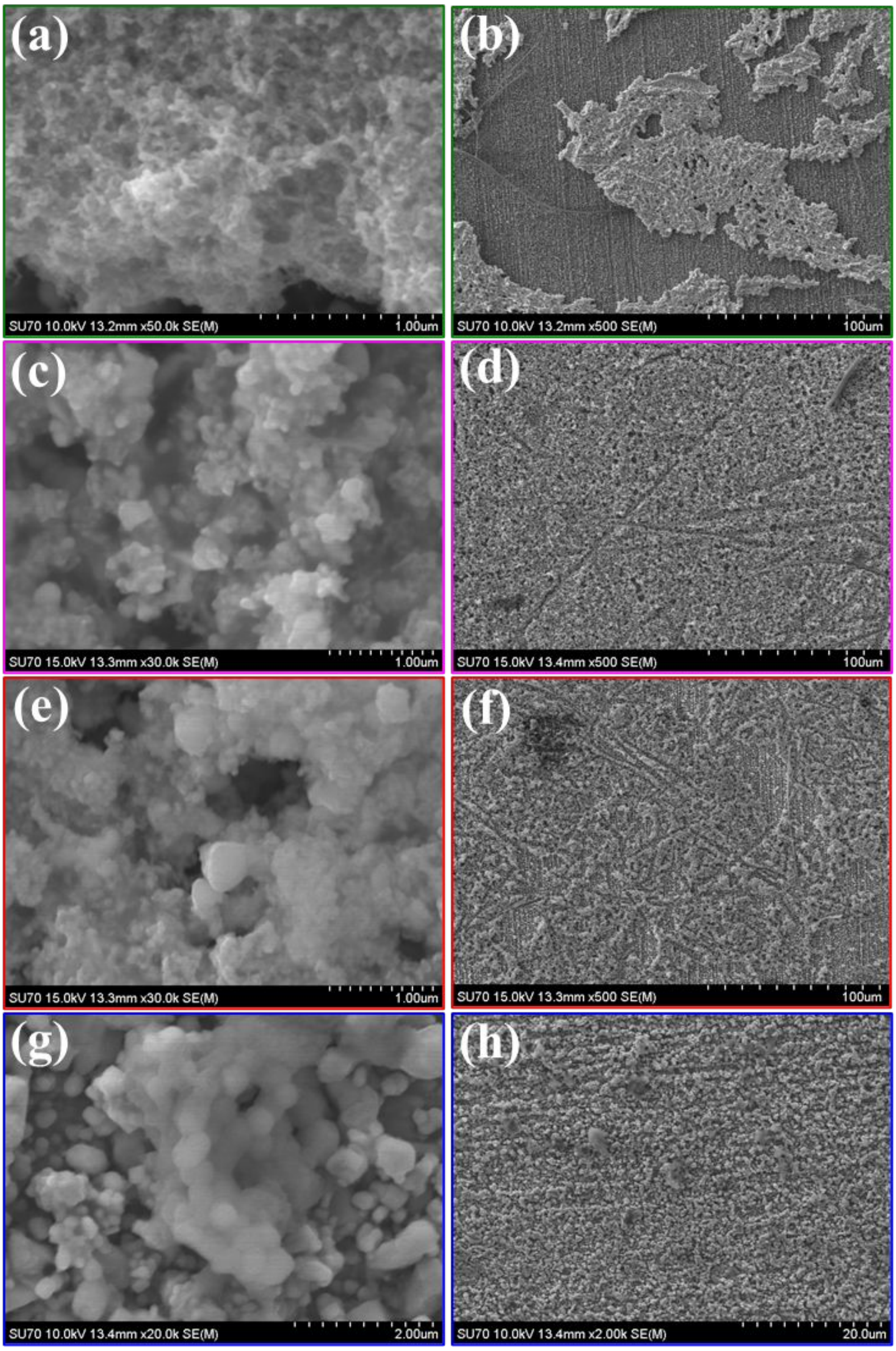

Figure S7 Structural morphology of (a-b) Bi_{100}, (c-d) $Bi_{66.5}Sn_{33.5}$ (e-f) $Bi_{50}Sn_{50}$, and (g-h) Sn_{100} electrodes after 200 cycles.

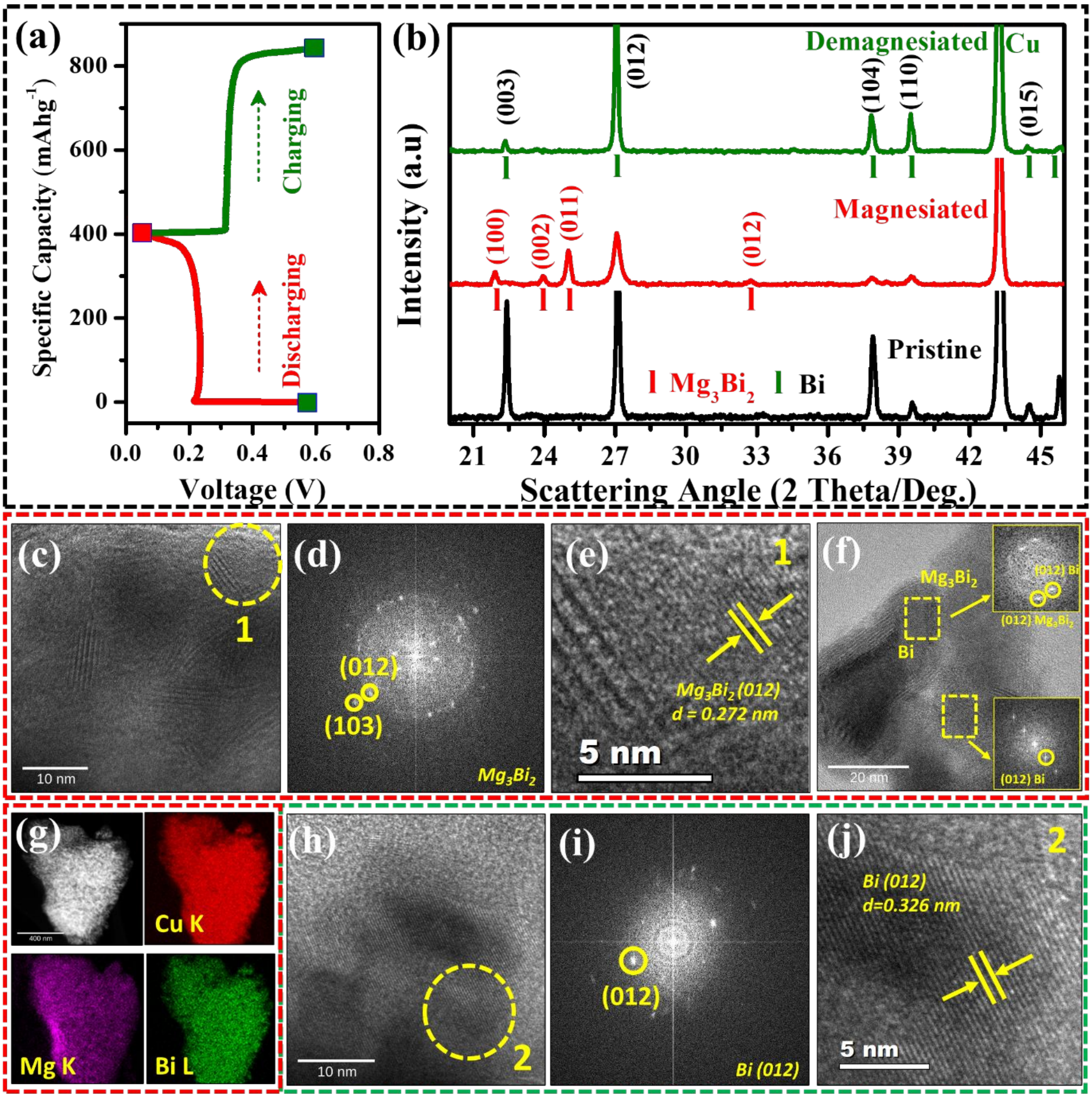

Figure S8 (a) Galvanostatic charging-discharging profile of Bi_{100} electrode, (b) XRD profiles of pristine Bi_{100} electrode, in magnesiated (discharged), and de-magnesiated (charged) states, (c) HRTEM image of magnesiated Bi_{100} electrode, and corresponding FFT shown in (d), (e) enlarged HRTEM image from circled area 1 in (c), (f) HRTEM image of electrode showing both Bi and Mg_3Bi_2 phases with insets showing corresponding FFTs, (g) STEM images of the electrode in magnesiated states, (h) HRTEM image of the electrode in de-magnesiated state and corresponding FFT shown in (i), and enlarged HRTEM image from circled area 2 in (h).

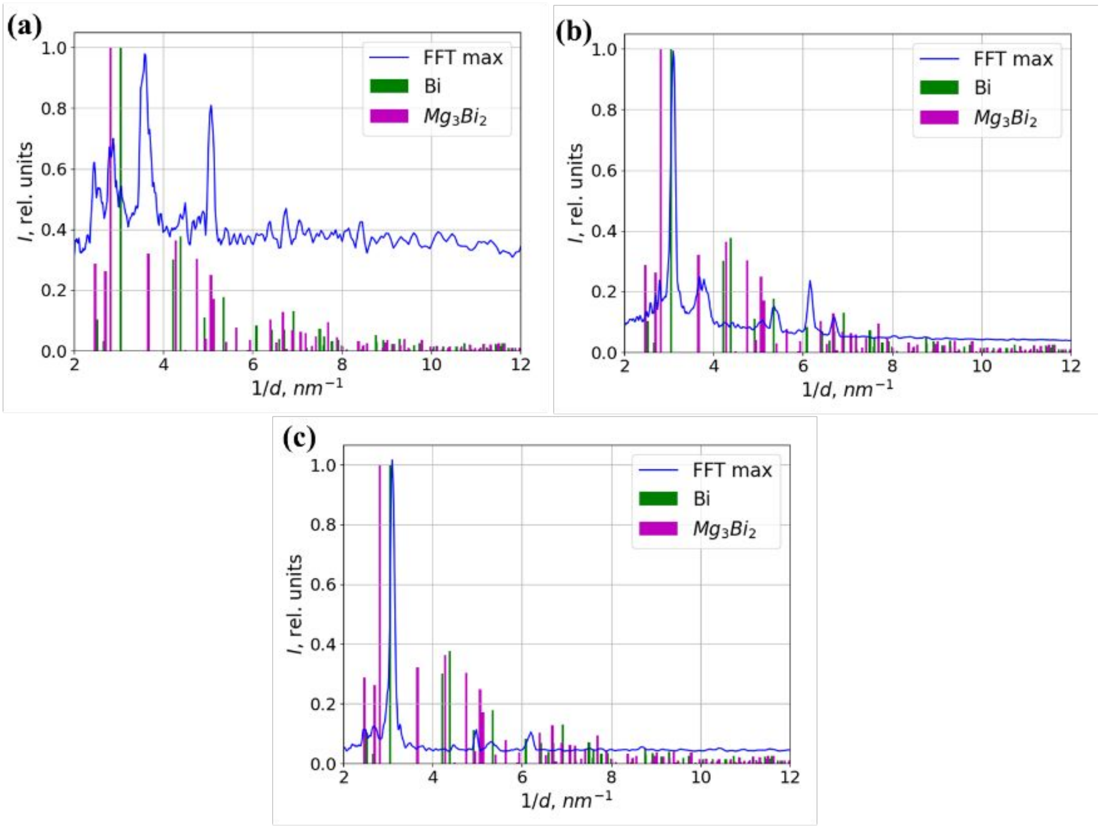

Figure S9 Integrated Fourier-transform of HRTEM images (Figure S9(a, b, c)) and bars corresponding to the Bi and Mg_3Bi_2 phases.

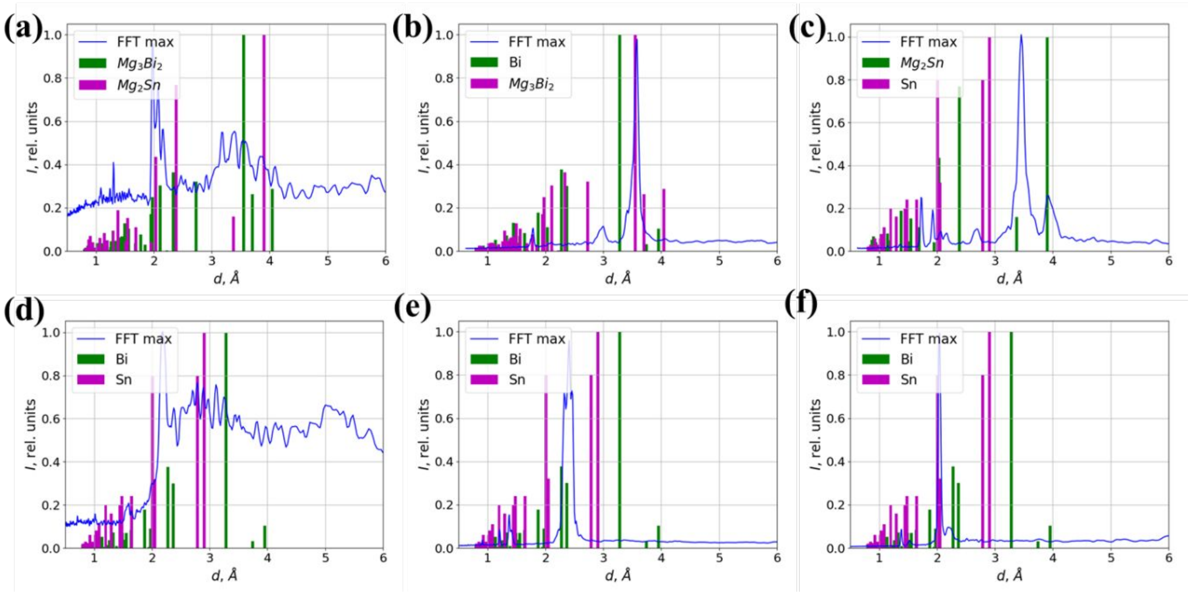

Figure S10 Integrated Fourier-transform of HRTEM images (Figure 6(b, d, c, j, k, l)) and bars corresponding to the Bi, Sn, Mg_3Bi_2 and Mg_2Sn phases.

Background to DFT calculations:

During the discharging process of the dual-phase Bi-Sn electrode, it was experimentally observed that Mg alloys with Bi before alloying with Sn. This prompted us to consider two relevant dual-phase interfaces: the fully magnesiated Bi//pure Sn and the fully magnesiated Bi//fully magnesiated Sn interfaces. The XRD characterizations performed in our study reveal specific active planes (hkl) for Sn, Bi, Mg_2Sn, and Mg_3Bi_2, as listed in **Table S1** (based on the experimental data also reported in **Figure 1j).**

Sn	Bi	Mg_2Sn	Mg_3Bi_2
(200)	(012)	(111)	(100)
(101)	(104)	(220)	(002)
	(110)		(011)
			(012)

Table S1 Different active planes (hkl) for Sn, Bi, Mg_2Sn, and Mg_3Bi_2 detected from XRD characterizations.

Based on these experimentally detected facets, there are 16 possible dual-phase models as follows:

- Fully magnesiated Bi//pure Sn dual-phase interfaces

  - Mg_3Bi_2(100)//Sn(200) and Mg_3Bi_2(100)//Sn(101)

  - Mg_3Bi_2(002)//Sn(200) and Mg_3Bi_2(002)//Sn(101)

  - Mg_3Bi_2(011)//Sn(200) and Mg_3Bi_2(011)//Sn(101)

  - Mg_3Bi_2(012)//Sn(200) and **Mg_3Bi_2(012)//Sn(101)**

- Fully magnesiated Bi//fully magnesiated Sn dual-phase interfaces

  - Mg_3Bi_2(100)//Mg_2Sn(111) and Mg_3Bi_2(100)//Mg_2Sn(220)

  - Mg_3Bi_2(002)//Mg_2Sn(111) and Mg_3Bi_2(002)//Mg_2Sn(220)

  - Mg_3Bi_2(011)//Mg_2Sn(111) and Mg_3Bi_2(011)//Mg_2Sn(220)

  - Mg_3Bi_2(012)//Mg_2Sn(111) and **Mg_3Bi_2(012)//Mg_2Sn(220)**

Given the scope of our study, which aimed to understand the action mechanism of the introduction of a second phase (*i.e.*, Mg_3Bi_2) and the Mg insertion process in the Bi-Sn dual-phase system, we focused on constructing the most relevant dual-phase interface (based on surfaces with the lowest surface energy, **Table S2**) rather than exploring all possible models.

To ensure the selection of appropriate interface models, we first calculated the surface energy (γ_{surface}) of all experimentally detected surfaces, as summarized in **Table S2**.

Table S2 Calculated surface (interface) energy (γ) in eV/Å^2 of different surfaces for Sn, Bi, Mg_2Sn, Mg_3Bi_2, and interface systems where their corresponding structures are shown in **Figure S13** for individual surfaces and **Figure S15** for interface systems.

Sn	γ_{Sn}	Bi	γ_{Bi}	Mg_2Sn	γ_{Mg_2Sn}	Mg_3Bi_2	$\gamma_{Mg_3Bi_2}$	Interface	$\gamma_{interface}$
(200)	**0.019**	(012)	**0.014**	(111)	**0.053**	(100)	**0.038**	Mg_3Bi_2(012)//Sn(101)	**0.006**
(101)	**0.018**	(104)	**0.021**	(220)	**0.027**	(002)	**0.039**	Mg_3Bi_2(012)//Mg_2Sn(220)	**0.050**
		(110)	**0.021**			(011)	**0.041**		
						(012)	**0.026**		

Table S2 Calculated surface (interface) energy (γ) in eV/Å^2 of different surfaces for Sn, Bi, Mg_2Sn, Mg_3Bi_2, and interface systems where their corresponding structures are shown in Figure S13 for individual surfaces and Figure S15 for interface systems.

The calculated surface energies indicate that Sn(101), Bi(012), Mg_2Sn(220), and Mg_3Bi_2(012) are the most stable surfaces. Consequently, only the Mg_3Bi_2(012)//Sn(101) and Mg_3Bi_2(012)//Mg_2Sn(220) interface models were chosen as they were built from the most stable individual surfaces and represent appropriate configurations, thus providing significant insights into the Mg insertion mechanism within the Bi-Sn dual-phase system. This rationale was discussed in the Computational Details section of our manuscript.

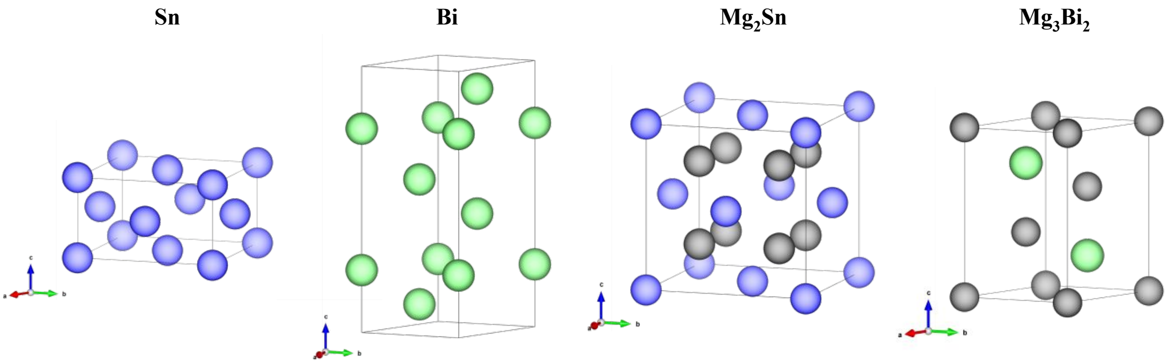

Figure S11 Optimized structures of the unit cells of Sn, Bi, Mg_2Sn, and Mg_3Bi_2, where the blue, green, and black balls represent Sn, Bi, and Mg atoms, respectively.

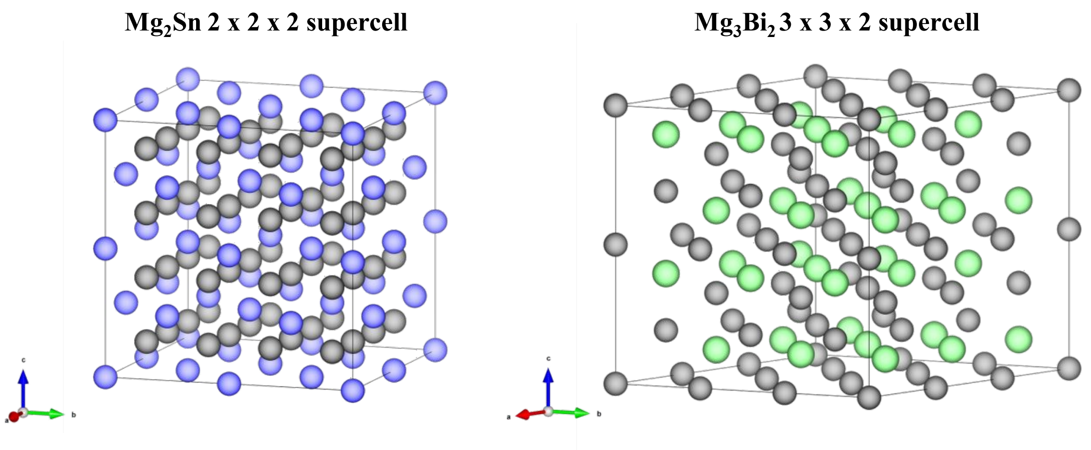

Figure S12 Optimized structures of Mg_2Sn 2×2×2 supercell and Mg_3Bi_2 3×3×2 supercell, where the blue, green, and black balls represent Sn, Bi, and Mg atoms, respectively.

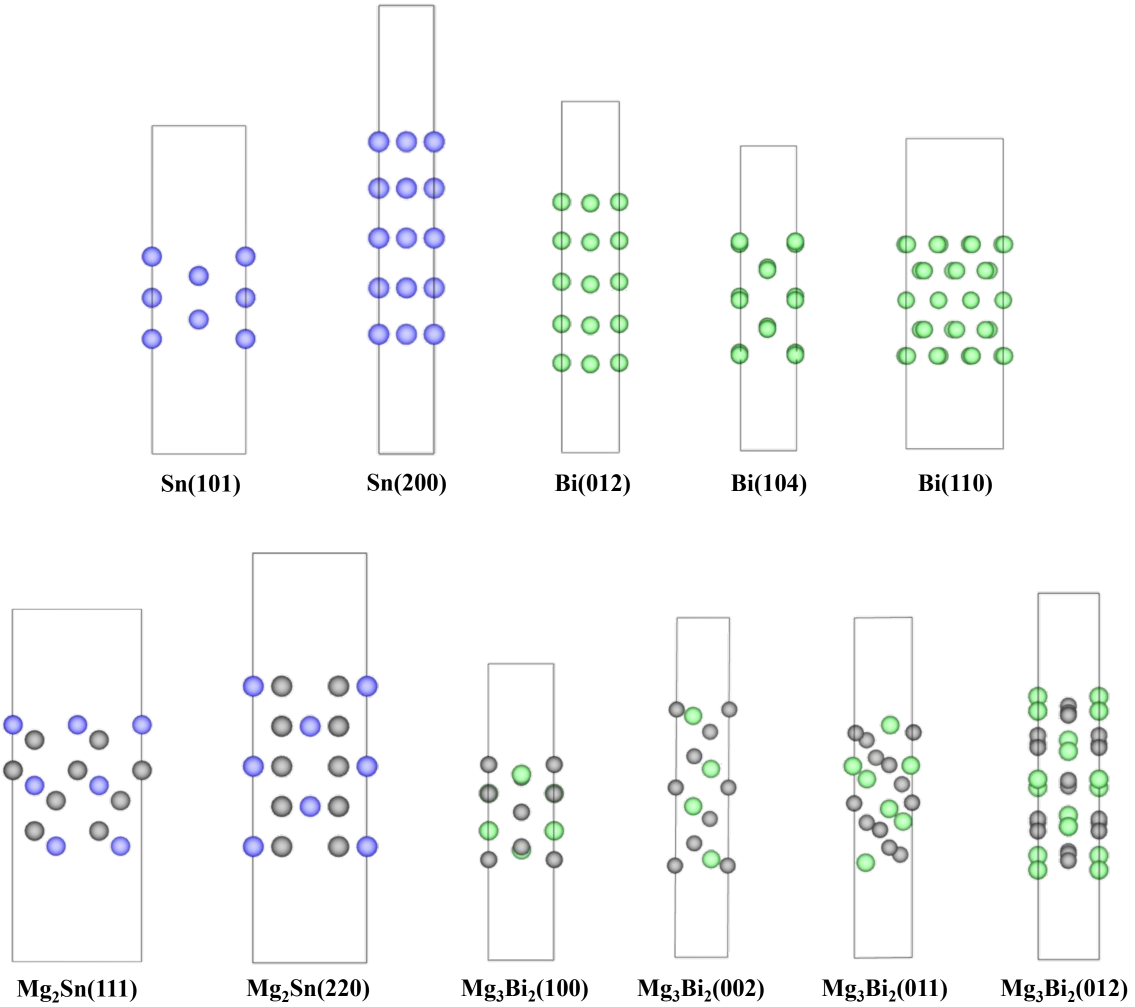

Figure S13 Different surfaces of Sn, Bi, Mg_2Sn, and Mg_3Bi_2 detected from XRD characterizations, where the blue, green, and black balls represent Sn, Bi, and Mg atoms, respectively.

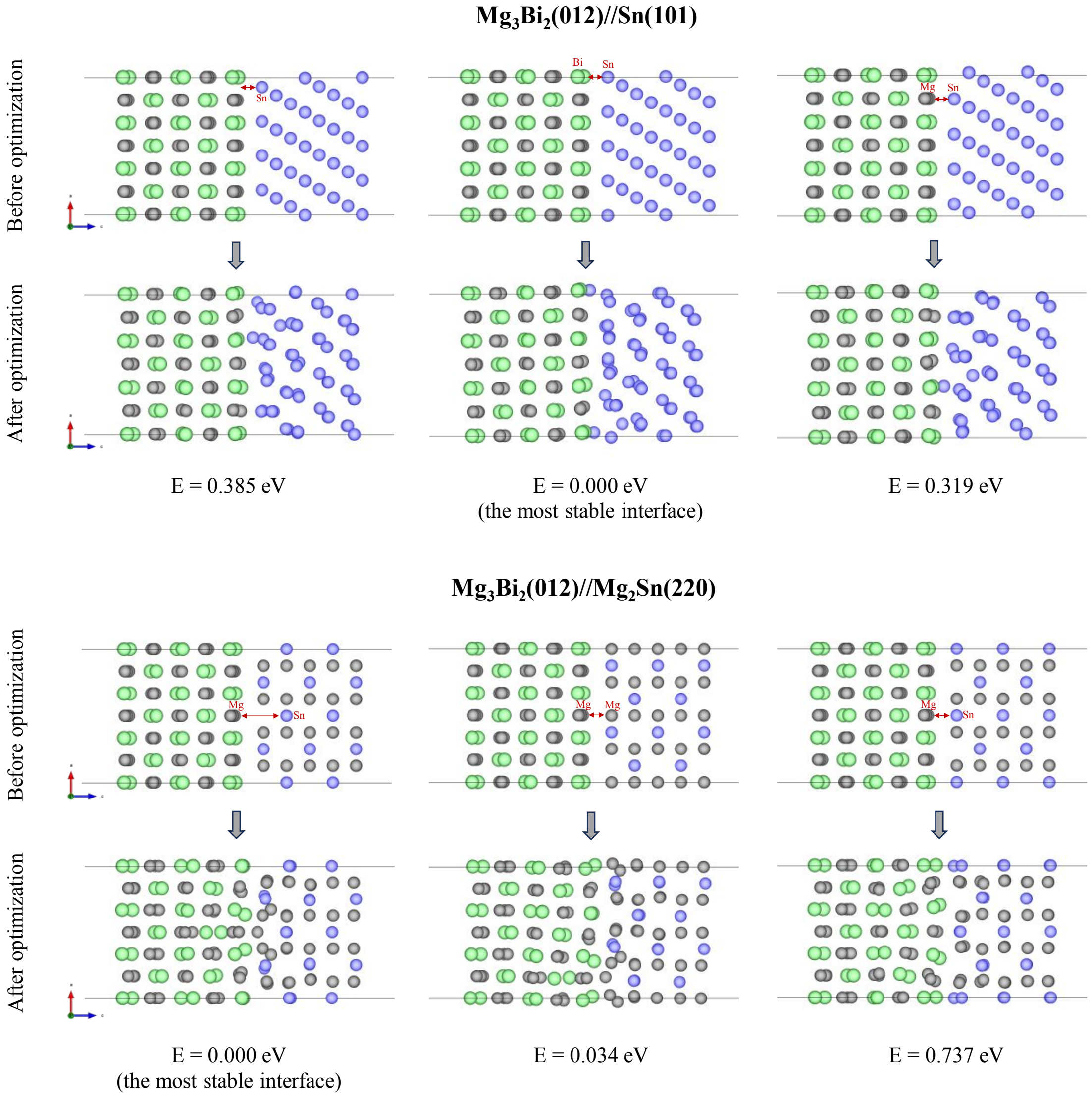

Figure S14 Different interface configurations of the dual-phase Mg_3Bi_2(012)//Sn(101) and Mg_3Bi_2(012)//Mg_2Sn(220), where the energy differences between different configurations are shown. The blue, green, and black balls represent Sn, Bi, and Mg atoms, respectively.

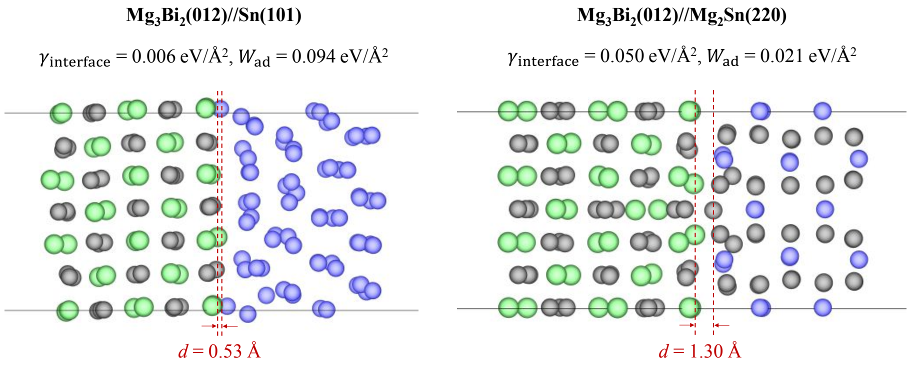

Figure S15 Optimized structures of the dual-phase $Mg_3Bi_2(012)//Sn(101)$ and $Mg_3Bi_2(012)//Mg_2Sn(220)$, where the interfacial distances (*d*) are shown which are calculated by the distance between the topmost surface atoms at the interface. The blue, green, and black balls represent Sn, Bi, and Mg atoms, respectively.

Table S3 The cell length changes of the pristine surfaces to form the dual-phase structures (Figure S15). The change in lattice parameters of the optimized interface compared to pristine supercell surface models is calculated.

Materials	*a* (Å)	*b* (Å)	*a* change (%)	*b* change (%)
3x1 $Mg_3Bi_2(012)$	13.846	10.829	+4.82	+1.27
2x2 Sn(101)	13.370	11.764	+8.55	−6.78
3x1 $Mg_3Bi_2(012)$// 2x2 Sn(101)	14.513	10.966	-	-
3x1 $Mg_3Bi_2(012)$	13.846	10.829	+0.16	−4.93
2x1 $Mg_2Sn(220)$	13.541	9.534	+2.42	+7.98
3x1 $Mg_3Bi_2(012)$// 2x1 $Mg_2Sn(220)$	13.868	10.295	-	-

Table S4 Cell parameters of all structures considered in this work and the corresponding k-points used for their geometry optimizations. The formation energy of bulk Mg_2Sn and Mg_3Bi_2 can be calculated as $\Delta E_{\mathrm{f}} = E_{\mathrm{Mg}_x\mathrm{M}_y} - xE_{\mathrm{Mg}} - yE_{\mathrm{M}}$, where $E_{\mathrm{Mg}_x\mathrm{M}_y}$, E_{Mg}, and E_{M} represents the total energies of the bulk Mg_xM_y, bulk Mg, and bulk M (M = Sn or Bi), respectively; x and y are the number of atoms of Mg and M in the bulk Mg_xM_y, respectively.

Materials	*a* (Å)	*b* (Å)	*c* (Å)	*α, β, γ* (°)	k-points
Bulk Sn unit cell	5.827	5.827	3.324	90, 90, 90	6×6×10
Bulk Bi unit cell	4.576	4.576	12.129	90, 90, 120	8×8×3
Bulk Sn 2×2×4 supercell	11.329	11.329	13.788	90, 90, 90	3×3×3
Bulk Bi 3×3×1 supercell	13.764	13.764	12.102	90, 90, 120	3×3×3
Bulk Mg_2Sn unit cell, ΔE_{f} = −0.226 eV/atom	6.820	6.820	6.820	90, 90, 90	5×5×5
Bulk Mg_3Bi_2 unit cell, ΔE_{f} = −0.216 eV/atom	4.708	4.708	7.456	90, 90, 120	8×8×4
Bulk Mg_2Sn 2×2×2 supercell	13.640	13.640	13.640	90, 90, 90	2×2×2
Bulk Mg_3Bi_2 3×3×2 supercell	14.123	14.123	14.911	90, 90, 120	3×3×2
3×1 $Mg_3Bi_2(012)$//2×2 Sn(101)	14.513	10.966	38.438	91.58, 91.92, 90.31	2×3×1
3×1 $Mg_3Bi_2(012)$//2×1 $Mg_2Sn(220)$	13.868	10.295	44.454	91.60, 90.07, 90	2×3×1

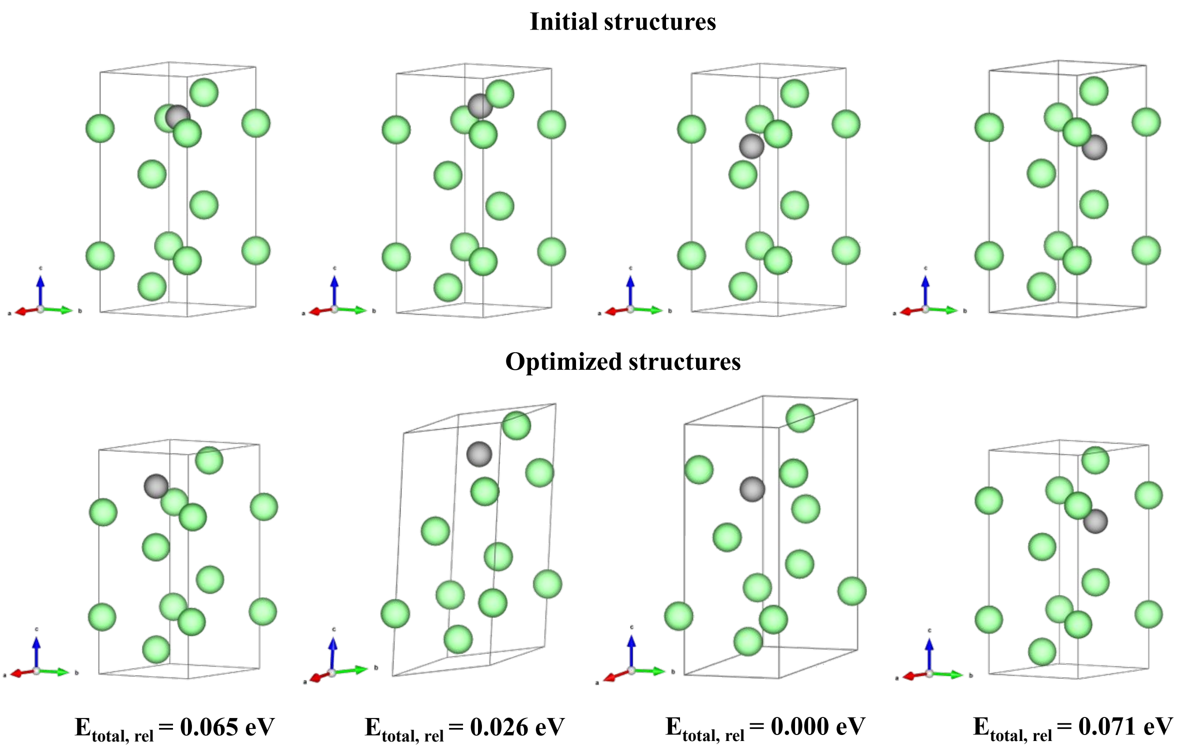

Figure S16 Different configurations of Mg insertion in Bi, where the initial structures and optimized structures are illustrated in the top and bottom panels, respectively. The green and black balls represent Bi and Mg atoms, respectively. The $E_{total,\ rel}$ terms denote the total energies of all considered configurations, which are referenced to the total energy of the most stable configuration.

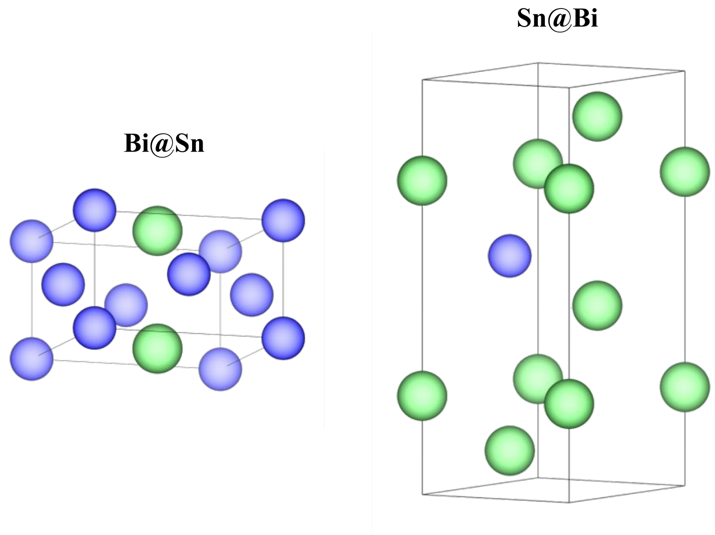

Figure S17 Optimized structures of a substituted Bi atom in Sn system and a replaced Sn atom in Bi system, where the blue and green balls represent Sn and Bi atoms, respectively.

Table S5 Calculated Mg vacancy formation energy in the bulk Mg_2Sn, bulk Mg_3Bi_2, and Mg_3Bi_2(012)//Mg_2Sn(220) interface models where different possible Mg vacancy sites were considered as shown in Figure S18. The Mg vacancy formation energy ($\Delta E_{\mathrm{f-Mg_{vac}}}$) can be calculated as $\Delta E_{\mathrm{f-Mg_{vac}}} = E_{\mathrm{system}} - E_{\mathrm{system,\ 1Mg_{vac}}} + E_{\mathrm{Mg}}$, where E_{system}, $E_{\mathrm{system,\ 1Mg_{vac}}}$, and E_{Mg} represent the total energy of the perfect system (bulk Mg_2Sn or bulk Mg_3Bi_2 or Mg_3Bi_2(012)//Mg_2Sn(220) interface), the total energy of the system with one Mg vacancy, and the total energy of a Mg atom, respectively.

System	Mg vacancy site	$\Delta E_{\mathrm{f-Mg_{vac}}}$ (eV)
bulk Mg_2Sn	Mg1	0.839
bulk Mg_3Bi_2	Mg1	0.996
	Mg2	1.101
Mg_3Bi_2(012)//Mg_2Sn(220) interface	Mg1	0.083
	Mg2	−0.356
	Mg3	0.091
	Mg4	0.095
	Mg5	0.106
	Mg6	0.254
	Mg7	0.306
	Mg8	0.256
	Mg9	0.210
	Mg10	0.504
	Mg11	−0.033
	Mg12	−0.368
	Mg13	−0.371
	Mg14	−0.354
	Mg15	−0.356
	Mg16	0.409
	Mg17	0.340

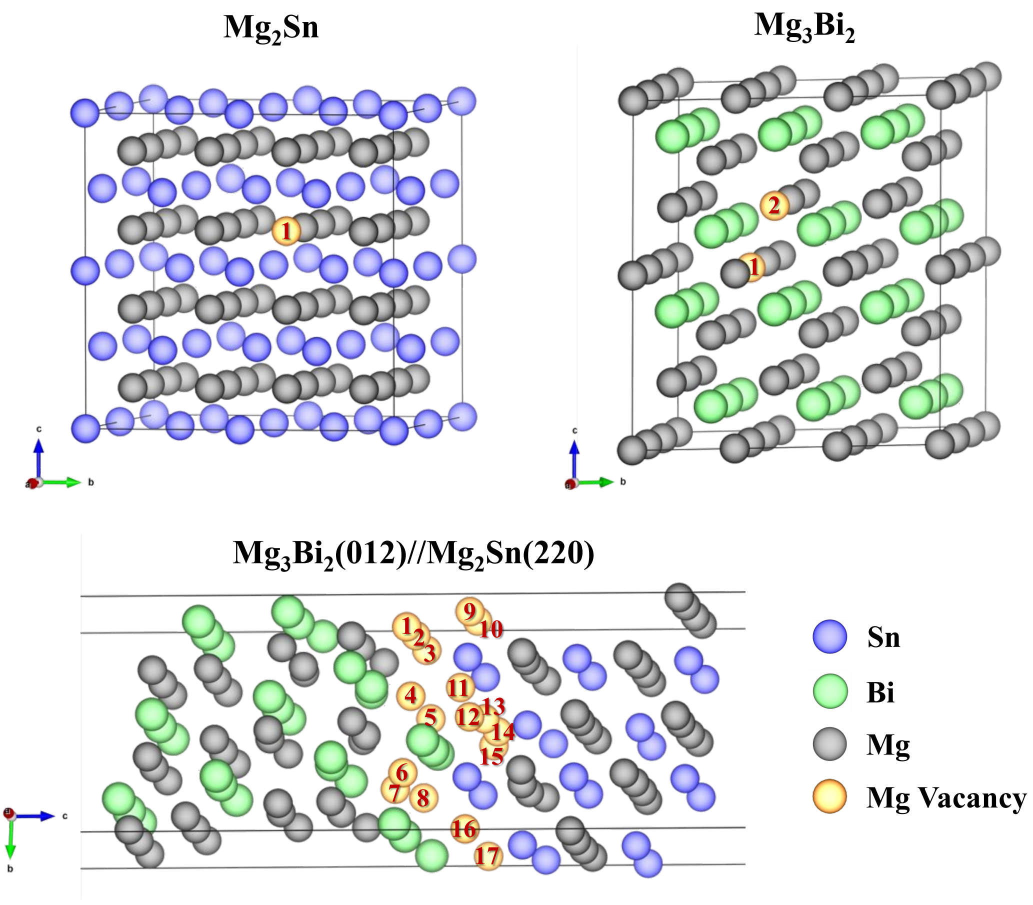

Figure S18 Different possible Mg vacancy sites considered in the bulk Mg_2Sn, bulk Mg_3Bi_2, and $Mg_3Bi_2(012)//Mg_2Sn(220)$ interface models.
